# Molecular and Serological Detection of Bovine Coronaviruses in Marmots (*Marmota marmota*) in the Alpine Region

**DOI:** 10.3390/v16040591

**Published:** 2024-04-11

**Authors:** Ana Moreno, Sabrina Canziani, Davide Lelli, Anna Castelli, Alessandro Bianchi, Irene Bertoletti, Federica Maccarinelli, Marco Carlomagno, Matteo Paini, Marzia Rossato, Massimo Delledonne, Stefano Giacomelli, Antonella Cordedda, Sandro Nicoloso, Enrica Bellinello, Anna Campagnoli, Tiziana Trogu

**Affiliations:** 1 Virology Unit, Istituto Zooprofilattico Sperimentale della Lombardia e dell’Emilia Romagna, Via A. Bianchi, 9, 25124 Brescia, Italy; sabrina.canziani@izsler.it (S.C.); davide.lelli@izsler.it (D.L.); anna.castelli@izsler.it (A.C.); f.maccarinelli@izsler.it (F.M.); tiziana.trogu@izsler.it (T.T.); 2 Sondrio Diagnostic Laboratory, Istituto Zooprofilattico Sperimentale della Lombardia e dell’Emilia Romagna, Via Bormio, 30, 23100 Sondrio, Italy; alessandro.bianchi@izsler.it (A.B.); irene.bertoletti@izsler.it (I.B.); 3 Department of Biotechnology, University of Verona, Strada le Grazie 15, 37134 Verona, Italy; marco.carlomagno@univr.it (M.C.); matteo.paini@univr.it (M.P.); marzia.rossato@univr.it (M.R.); massimo.delledonne@univr.it (M.D.); 4 Genartis s.r.l., Via IV Novembre 24, 37126 Verona, Italy; 5 ATS della Montagna, Via Nazario Sauro, 36/38, 23100 Sondrio, Italy; st.giacomelli@ats-montagna.it (S.G.); antonella.cordedda@yahoo.it (A.C.); 6 Dimensione Ricerca Ecologie e Ambiente Italia Società Cooperativa, Via Enrico Bindi n. 14, 51100 Pistoia, Italy; nicoloso@dream-italia.it (S.N.); enrica.bellinello@studio.unibo.it (E.B.); 7 ATS Insubria, Via Ottorino Rossi, 9, 21100 Varese, Italy; anna.campagnoli@virgilio.it

**Keywords:** marmots, Alpine region, bovine coronavirus, whole genome sequencing, serology

## Abstract

In this study, virological surveillance focused on coronaviruses in marmots in the Alpine region in 2022, captured as part of a population control reduction program in the Livigno area. Seventy-six faecal samples were randomly collected from marmots at the time of capture and release and tested for genome detection of pan-coronavirus, pan-pestivirus, canine distemper virus, and influenza A and D virus. Nine faecal samples were positive in the Pan-CoV RT-PCR, while all were negative for the other viruses. Pan-coronavirus positives were further identified using Illumina’s complete genome sequencing, which showed the highest homology with Bovine Coronavirus previously detected in roe deer in the Alps. Blood samples (n.35) were collected randomly from animals at release and tested for bovine coronavirus (BCoV) antibodies using competitive ELISA and VNT. Serological analyses revealed that 8/35 sera were positive for BCoV antibodies in both serological tests. This study provides molecular and serological evidence of the presence of BCoV in an alpine marmot population due to a likely spillover event. Marmots share areas and pastures with roe deer and other wild ruminants, and environmental transmission is a concrete possibility.

## 1. Introduction

Coronaviruses (CoVs) belong to the order Nidovirales, family Coronaviridae, and have a positive-sense, non-segmented, single-stranded RNA genome of about 27–32 Kb. CoVs are characterised by high antigenic and genetic variability and evolve continuously, often being able to cross the host barrier and cause spillover events. Several CoVs have emerged in humans and animals in the past two decades, causing considerable economic and human life losses. The reporting of three highly pathogenic human coronaviruses (SARS-CoV, MERS-CoV, and SARS-CoV-2) and, in particular, the recent epidemic of COVID-19 have raised great concern and interest in better understanding spillover events and interspecies transmission in coronaviruses. It is, therefore, crucial to identify their natural reservoirs and the circumstances under which their transmission can occur. 

The subfamily *Orthocoronavirinae* is currently classified into four genera: *Alphacoronavirus* (α-CoV), *Betacoronavirus* (β-CoV), *Gammacoronavirus* (γ-CoV), and *Deltacoronavirus* (δ-CoV) (https://talk.ictvonline.org/taxonomy/, accessed on 10 January 2024) [1]. Members of the first two genera (α and β-CoV) infect mammals, while members of the other two mainly infect avian species [2]. In particular, the *Embecovirus* subgenus within β-CoVs includes murine hepatitis virus (MHV), bovine coronavirus (BCoV), human coronavirus OC43 (HCoV-OC43), porcine haemagglutinating encephalomyelitis virus (PHEV), and canine respiratory coronavirus (CRCoV). Unlike other alpha- and betacoronavirus species, these viruses do not seem to have ancestral links with bats but with rodents, as suggested by several studies [2,3]. Rodents are important reservoirs for members of this subgenus, with a likely key role in their evolution and emergence. Out of these viruses, BCoV is an excellent example of a CoV that largely crosses the interspecific barrier. Several bovine-like CoVs have been identified as agents of respiratory or enteric diseases in a wide range of ruminant species [4], dogs [4,5], and even humans [6]. It has been hypothesised that the presence of the haemagglutin-esterase protein, which allows the virus to bind to different cell types, determines BCoV’s broad host spectrum [4].

The Alpine marmot (*Marmota marmota*) is a rodent belonging to the *Sciuridae* family. It lives at altitudes above 1500 m, at the upper edge of the forest, where the trees thin out and decrease in size. Typically, the altitudes at which the marmot lives range from 2000 to 3000 m. In summer, they live in large burrows they have dug themselves, providing shelter for the whole family. At the same time, when the first cold weather arrives, they migrate to lower altitudes, digging burrows that they will fill with tender grasses and roots and whose entrance they will obstruct to prevent excessive winter cooling [7]. The Alpine marmot is the largest rodent in the Alpine region and predominantly occupies grassland environments between 600 and 3200 m above sea level. In the Livigno province, the marmot population and those of various species of small rodents and insectivores reach high densities. In addition, wild ruminants are permanently present in the same area, such as the red deer (*Cervus elaphus*), the roe deer (*Capreolus capreolus*), the Alpine chamois (*Rupicapra rupicapra*), and the Alpine ibex (*Capra ibex*). The Alpine marmot forms monogamous families consisting of a dominant male, a single reproductive female, and possibly the young, sub-adults, and other adults playing the role of helpers [7]. Family cohesion is maintained through certain social behaviours, such as mutual cleaning, playing, and contact between the muzzles of individuals. 

The municipality of Livigno, in the Eastern Alps, has experienced a significant increase in the marmot population in recent years, which has resulted in considerable damage to the area’s meadows and pastures. For this reason, a project was undertaken to address the problem of the marmot’s impact on the area’s mountain pastures in compliance with current regulations. This project involved capturing a defined number of specimens and transferring them from Livigno to the Adamello and Alto Garda regional parks (Brescia province) in the Eastern Alps but at a lower altitude. In fact, in these areas, this species has disappeared for years, causing damage to the entire ecosystem. The project has, therefore, made it possible to solve the problem in Livigno and, at the same time, reintroduce marmots where they have been absent for some time. The initiative was taken after an in-depth naturalistic study that considered the main environmental variables (altitude, sun exposure, slopes, and vegetation present). This project, therefore, represented an excellent opportunity for sample collection and virological surveillance in this little-studied species.

Indeed, this study reports on the virological surveillance carried out in 2022 in marmots captured, transferred, and released as part of the project, focusing on detecting coronaviruses. Bovine coronavirus detection was evidenced using both molecular and serological methods, showing a species jump and a spillover event. Coronavirus surveillance in wild hosts is essential to better understand viral diversity and may also provide valuable indications to reduce the risk of future spillover events from animals to humans.

## 2. Materials and Methods

### 2.1. Sampling

**Figure 1:**
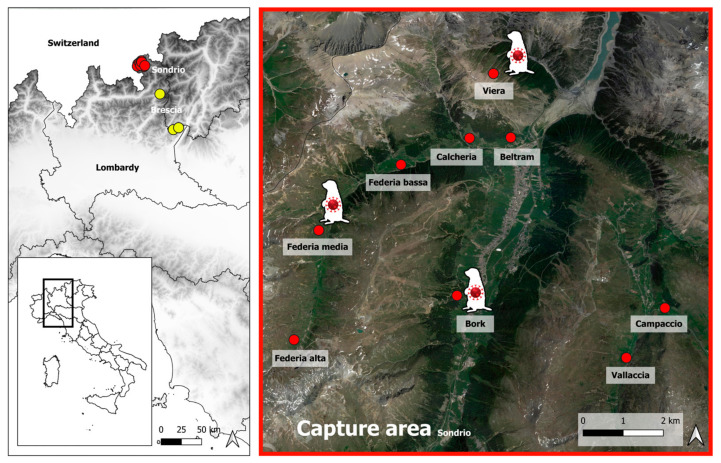
Geographical location of the capture (red) and release (yellow) areas. Capture areas where coronavirus-positive animals were found are indicated.

As part of the provincial relocation plan implemented in 2022, alpine marmots were captured in different areas of the municipality of Livigno in Sondrio province (northern Italy). After a few days of quarantine, they were released to other alpine locations. Each captured animal was measured and checked for the presence of clinical signs. Animal sampling was not explicitly considered in this plan, but to better investigate the health status of the animals, some marmots were randomly sampled by collecting faecal samples. Sixty animals were sampled during capture (1st sampling) in Livigno province. To further investigate the results observed at the first sampling and given the availability of an official veterinarian, it was decided to collect the blood from a further 35 marmots and faeces from 16 animals randomly sampled at the time of release a few days later (2nd sampling) in some areas of the neighbouring Brescia province. When possible, the family social structure was ensured during the relocation process. During the sampling, the animals were in good health and showed no signs of disease. Data on the areas and dates of capture and release, as well as the sex, age, body measurements, and samples collected from each animal, are reported in Table S1. The areas of capture and release and the capture sites with coronavirus-positive marmots are shown in Figure 1.

### 2.2. Diagnostic Examinations

Viral RNA from faecal samples was extracted using the QIAsymphony SP instrument and QIAsymphony DSP Virus/Pathogen Kit, Qiagen GmbH, Qiagen Strasse 1, 40724 Hilden, Germany, according to the manufacturer’s instructions. Samples were then tested for coronaviruses using a nested Pan-Coronavirus RT-PCR (Pan-CoV) targeting the RNA-dependent RNA gene (RdRp) and a real-time RT-PCR for SARS-CoV-2 performed as described [8,9,10]. PCR-positive samples for Pan-CoV were sequenced using Sanger sequencing technology. Moreover, all samples were tested using different real-time RT-PCR methods for the detection of pan-pestiviruses [11], canine distemper virus (CDV) [12], and influenza A [13] and D [14] viruses. 

### 2.3. Virus Isolation

Virus isolation was attempted by inoculating Pan-CoV PCR-positive faecal samples into different cell lines, such as VERO (African green monkey kidney), MARC-145 (fetal monkey kidney), and HRT-18 (human colorectal adenocarcinoma) cells. Twenty-four-well tissue culture plates were inoculated with 0.2 mL of clarified pathological material per well. After adsorption for 1 h at 37 °C, maintenance medium (0.8 mL per well) was added without removing the viral inoculum, and the cultures were incubated at 37 °C with 5% CO_2_ and observed daily for 5–6 days. Three blind passages were performed before the sample was discarded as negative.

### 2.4. Metatranscriptome Sequencing and Phylogenetic Analysis

RNA integrity and concentration were assessed using the RNA 6000 Pico kit on a Bioanalyzer 2100 (Agilent Technologies, Santa Clara, CA, USA). A total of 1.3 ng RNA was treated with the Heat&Run gDNA removal kit (ArcticZymes, Tromsø, Norway) and subsequently with the Pan-Prokaryote riboPOOL kit (SiTOOLS Biotech, Planegg, Germany), according to the manufacturers’ instructions, to remove residual DNA and prokaryotic rRNA, respectively. RNA-seq libraries were generated from the whole amount of RNA recovered after the previous step using the SMARTer Stranded Total RNA-Seq kit v3 (Takara Bio, Shiga, Japan), including a mammalian rRNA depletion step. Given the high degradation and low input, RNA was not fragmented, and libraries were generated according to the Pico Input protocol described by the manufacturer. RNAseq libraries were quantified using real-time PCR against a standard curve with the KAPA Library Quantification Kit (Kapa-Biosystems, Wilmington, MA, USA) and sequenced in 150PE mode on a NovaSeq6000 sequencer (Illumina, San Diego, CA, USA), generating an average of 80 million fragments per sample. Raw reads were trimmed with cutadapt (v.2.8) (https://doi.org/10.14806/ej.17.1.200, accessed on 14 November 2023), with parameters -m 15 -u 3. Reads from rRNA fragments were identified with RiboDetector (v.0.2.7) (https://doi.org/10.1093/nar/gkac112, accessed on 14 November 2023) and subsequently excluded from the analysis. Residual reads belonging to the host were identified by mapping the data on the marmot reference genome (Alpine marmot—GCF_001458135.2) with STAR (v2.7.11a) (https://doi.org/10.1093/bioinformatics/bts635, accessed on 25 October 2012). The unmapped reads were assembled with MEGAHIT (v1.2.9) (https://doi.org/10.1093/bioinformatics/btv033, access on 20 January 2015), with a minimum contig length of 200. The assembled contigs were blasted against the RefSeq Viral database with BLASTn (v.2.9.0+) (https://doi.org/10.1016/S0022-2836(05)80360-2, access on 6 February 2007). The alignment of nucleotide and amino acid sequences and the calculation of pairwise identities of genomes were performed using MEGA 11v.11.0.11, and LASERGENE DNASTAR software, v.17.3.0.57. The Open Reading Frames (ORFs) were predicted using the online ORF Finder tool (NCBI, http://www.ncbi.nlm.nih.gov/gorf/gorf.html, accessed on 9 February 2024).

A dataset of complete genomic sequences of BCoV and other related β-CoVs for phylogenetic analyses was set up, with sequences obtained from the NIAID Virus Pathogen Database and Analysis Resource (ViPR) via the website http://www.viprbrc.org/ (accessed on 9 February 2024). Multiple sequence alignment was calculated using the MUSCLE algorithm. A maximum likelihood phylogenetic tree was performed using IQtree (2.3.1) [15] and Model finder software (2.3.1) to determine the best model based on the BIC [16]. Genetic relationships between Italian Marmot-CoVs, BCoV, and human OC43-CoV were investigated by generating similarity plots of these genomes by using SSE v1.2 with a sliding window of 600 and a step size of 100 nucleotides (nt), available at http://www.virus-evolution.org/Downloads/Software/ (accessed on 9 February 2024) [17].

Molecular analysis of the two main proteins (Spike (S) and haemagglutinin-esterase (HE) proteins) was performed by comparing the two Italian marmot sequences with a dataset of bovine coronavirus sequences available from the GenBank database, including reference B-CoV strains and closely related strains originated from France and Ireland. Alignment was performed using the ClustalW method in the MegaX 7.0 software. The N-linked glycosylation sites were predicted with the NetNGlyc-1.0 software (https://services.healthtech.dtu.dk/services/NetNGlyc-1.0/, accessed on 25 March 2023).

### 2.5. Antibody Detection against BCoV

Sera were analysed with an in-house MAb-based competitive ELISA developed for the detection of antibodies against bovine coronavirus (BCoV) at IZSLER MP04/143 rev01 [5]. This ELISA is based on the use of the BCoV strain 9WBL77 captured by a BCoV-specific polyclonal guinea pig serum coated to the plate. The reaction involves the addition of a BCoV-specific MAb competing with antibodies in tested sera for antigen binding. Briefly, (i) a polyclonal guinea pig anti-BCoV serum is coated to the plate (50 µL/well) to capture the antigen obtained by the propagation of BCoV on HRT-18 cultured in Dulbecco’s minimal essential medium (D-MEM) supplemented with 10% foetal calf serum and then inactivated with beta-propiolactone. (ii) After being stored overnight at 4 °C, BCoV antigen is added to the plate (50 µL/well). (iii) Following incubation at 37 °C for 1 h, 50 µL of serum samples tested in four eight-fold dilutions from 1/50 to 1/25,600 is added, including negative and positive controls in each plate. (iv) Following incubation at 37 °C for 1 h, 25 µL of the HRP-conjugated anti-BCoV MAb is added to the plate. Results are expressed as % inhibition versus 100% according to the formula: % of inhibition = 100 − (Optical Density (OD) serum/OD 100%) × 100. Sera were considered positive with ≥75% inhibition at the first dilution.

Since the MAb-based competitive ELISA was set up for cattle sera, another serological method was employed to confirm the ELISA-positive sera. The golden standard test Virus-Neutralisation assay (VNT) was performed as described [18] with minor changes. Briefly, the sera were heat-inactivated at 56 °C for 30 min. The BCoV strain was diluted to obtain 100 TCID_50_ per inoculum. An equal volume of serial dilution serum samples (eight two-fold serial dilutions starting from 1/5) and 100 TCID_50_ virus suspension were mixed and incubated at 37 °C for one hour before adding 50 µL in each well of 96-well tissue culture plates. Cellular and virus control were also included. A cellular suspension was prepared and added to all wells, and the plates were incubated at 37 °C, 5% CO_2_, for four days with daily microscopic examination for cytopathic effects. The endpoint was the final serum dilution that completely inhibited the viral cytopathic effects in both wells. 

## 3. Results

### 3.1. Diagnostic Examinations

All marmot faecal samples yielded negative results for pan-pestiviruses, CDV, influenza A, D, and SARS-CoV-2 virus genomes. Interestingly, nine faecal samples collected during the first sampling yielded positive results in the Pan-CoV RT-PCR (Table S1).

Coronavirus-positive samples were inoculated to cell cultures without any success in virus isolation. These samples were named as follows: Marmot coronavirus/Italy/193728-33/2022 (Marmot-CoV-33); Marmot coronavirus/Italy/193728-34/2022 (Marmot-CoV-34); Marmot coronavirus/Italy/193728-35/2022 (Marmot-CoV-35); Marmot coronavirus/Italy/193728-36/2022 (Marmot-CoV-36); Marmot coronavirus/Italy/193728-53/2022 (Marmot-CoV-53); Marmot coronavirus/Italy/193728-56/2022 (Marmot-CoV56); Marmot coronavirus/Italy/193728-57/2022 (Marmot-CoV-57); Marmot coronavirus/Italy/193728-58/2022 (Marmot-CoV-58); and Marmot coronavirus/Italy/193728-59/2022 (Marmot-CoV-59). They correspond to the marmot identificatory numbers 246, 249, 250, 251, 332, 336, 339, 340, and 341, respectively. 

### 3.2. Genome Sequencing and Phylogenetic and Molecular Analysis

**Figure 2:**
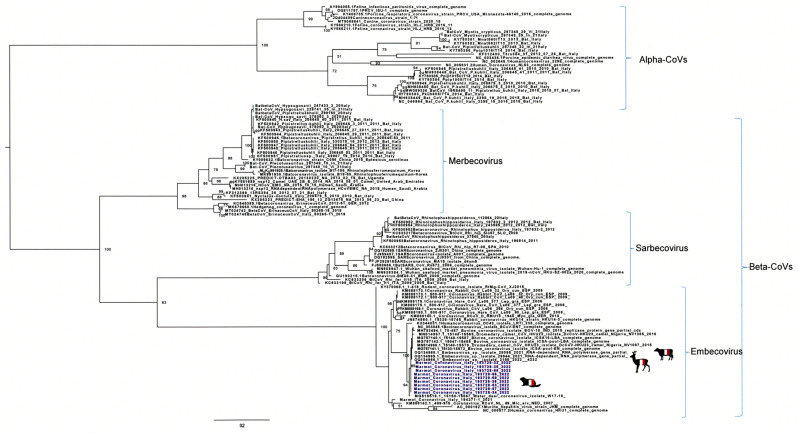
Maximum-likelihood phylogenetic tree based on the RdRp partial sequences from the Italian marmots. RdRp genome sequences of alpha- and betacoronaviruses are included. Sequences are identified with GenBank accession number, host, strain name, year of isolation, and country. Italian marmot sequences are highlighted in blue. GenBank accession numbers for the Italian marmot sequences are as follows: Marmot-CoV-33 (PP534466, partial RdRp gene sequence); Marmot-CoV-34 (PP534473, complete genome sequence); Marmot-CoV-35 PP534474, complete genome sequence); Marmot-CoV-36 (PP534467, partial RdRp gene sequence); Marmot-CoV-56 (PP534468, partial RdRp gene sequence); Marmot-CoV-56 (PP534469, partial RdRp gene sequence); Marmot-CoV-57 (PP534470, partial RdRp gene sequence); Marmot-CoV-58 (PP534471, partial RdRp gene sequence); and Marmot-CoV-59 (PP534472, partial RdRp gene sequence). Italian marmot sequences are highlighted in blue.

The partial sequencing of the nine samples positive in the Pan-CoV RT-PCR targeting the RpRd gene showed 100% of homology between them. Blast analysis showed the highest homology (99.71%) to four sequences of *Embecovirus* sp., BCoV, which were detected in roe deer (*Capreolus capreolus*) in the Alpine Mountains in Italy [19]. The phylogenetic analysis of the partial RdRp sequences from the Italian marmots confirmed the close relationship with the Italian roe deer’s sequences (Figure 2). 

Out of these samples, two (Marmot-CoV-34 and Marmot-CoV-35) were selected to be fully sequenced using metatranscriptome sequencing, which produced 26.3 and 23.6 Gb per sample, respectively. De novo assembly generated a total of 2903 contigs (max length = 202,033 bp, N50 = 37,361 bp) for sample Marmot-CoV-34 and 2972 contigs (max length = 288,352 bp, N50 = 36,459 bp) for Marmot-CoV-35. Two contigs of 31,231 bp (Marmot-CoV-34) and 31,183 bp (Marmot-CoV-35) matched with best-hits classified as “Coronavirus” after blast analysis against the RefSeq Viral database. Complete coronavirus sequences obtained from marmots were assembled with the NCBI reference sequence of BCoV-ENT (GenBank accession number NC00345). The genome structure of Marmot-CoV34 and Marmot-CoV35 is identical to that of BCoV, with the following genome organisation: 5′ untranslated region (UTR)-ORF1a (polyprotein 1a)-ORF1b (polyprotein 1b)-ORF2 (32 kDa protein)-ORF3 (hemagglutinin esterase)-ORF4 (spike protein)-ORF5 (4.9 kDa protein)-ORF6 (4.8 kDa protein)-ORF7 (12.4 kDa protein)-ORF8 (small membrane protein)-ORF9 (membrane protein)-ORF10 (nucleocapsid protein)-ORF11 (internal protein)-3′ UTR. The two full genome sizes comprised 31,022 nt with a G+C content of 36% each and showed 99.92% identity between them. 

**Figure 3:**
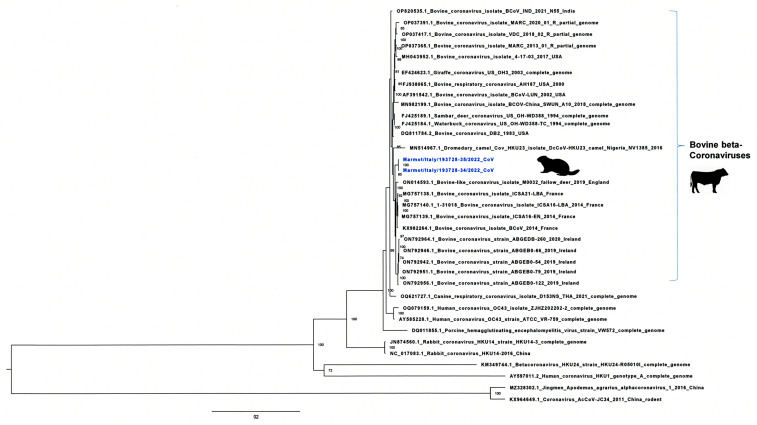
Maximum-likelihood phylogenetic tree based on the whole genome sequences from the Italian marmots. Complete genome sequences of *Embecoviruses* within β-CoVs from ruminants, pig, dogs, and humans are included. Sequences are identified with GenBank acc number, host, strain name, year of isolation, and country. Italian marmot sequences are highlighted in blue.

BLASTN analysis revealed that the two full genome sequences showed the highest nucleotide identity (98.88% and 98.89%, respectively, for Marmot-CoV34 and 35) with the BCoV strain ICSA16-EN (GenBank accession number MG757139), detected in 2014 from a bovine in France. Whole-genome-based phylogenetic analysis confirmed these results and showed that the two Italian genomes belong to the *Embecovirus* subgenus and are closely related to bovine coronavirus sequences from cattle circulating in France and Ireland (Figure 3).

**Figure 4:**
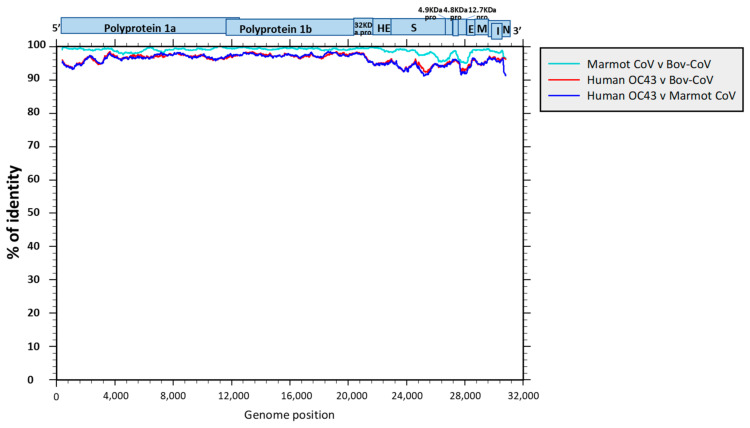
SEE similarity plots over the whole genome sequences between the Italian marmot, BCoV, and human OC43 sequences. The genome structure of marmot coronaviruses is reported.

The genomic identity of the marmot sequences with respect to BCoV and human OC43 CoV is shown in Figure 2, confirming the high percentage of identity, greater than 90% over the whole genome, between the marmot sequences and the French BCoV sequence (Figure 4).

The blast analysis of all Marmot-CoV-35 coronavirus proteins showed high percentages of identity, from 96.55 to 100%, with BCoV proteins (Table 1).

A molecular analysis of the complete S-protein of Italian marmots was performed in comparison to reference B-CoV and closely related field B-CoV strains from Europe. No deletions or insertions were observed in the Italian sequences. The comparison of amino acid sequences showed few aa changes in the S-proteins of Marmot-34 (no.43) and 35 (no.42) compared to the reference Mebus strain. Amino acid changes were reduced to only 19 compared to the S-proteins of the most closely related Irish B-CoVs and of these, 7 mutated aa were found only in marmots but not related to the sites of interest (Table S2). The S-proteins of the Italian marmots and related Irish and French B-CoVs contain 21 N-glycation sites at the aa positions 59, 133, 138, 198, 359, 437, 444, 649, 676, 696, 714, 739, 788, 895, 937, 1194, 1224, 1234, 1253, 1260, 1267, and 1288, 1 more (1260) than in the Mebus strain. No interesting changes of aa were observed in the HE protein of Marmot CoVs compared to the more related proteins of B-CoVs from France and Ireland (two changes of aa in Marmot-34 and one in Marmot-35) (Table S3).

### 3.3. Serologic Analyses

A serological investigation was considered to confirm the Pan-CoV genome detection results. Serum samples collected from marmots before release were initially tested using the BCoV MAb-based competitive ELISA, which detected 8/35 positive samples, all except 1 with a high inhibition percentage (≥90%). However, this ELISA, although a competitive ELISA capable of testing sera from different animal species, was specifically developed and validated to test sera from ruminants. Since no information about its use with marmot sera and its relative optimal cut-off value was available, all marmot sera were also tested with the BCoV VNT to validate the results obtained. VNT assays confirmed seven samples positive at different VNT titers from 1/10 to 1/80 (Table 2). Only one ELISA-positive sample was VNT-negative, but it was the one with the lowest inhibition percentage. The values shown in Table 2 show the concordance between the positive or negative qualitative results detected using the two methods and confirm the positivity detected using ELISA with VNT as the golden standard test. However, it has to be considered that the types of antibodies detected with the two methods are different, and therefore a comparative evaluation of the quantitative results detected with both methods cannot be made.

## 4. Discussion

The project undertaken by the Municipality of Livigno, in agreement with the Province of Sondrio and the Mountain Community of Aviolo, Edolo Basin, in the Province of Brescia, to transfer and relocate marmots provided an excellent opportunity to investigate the health status of this species, about which very little is known. The collection of faeces and blood samples made it possible to investigate the presence of some viruses, such as influenza A and D viruses, canine distemper virus, and coronaviruses, which may be of interest for their zoonotic potential and/or for their consolidated pathogenicity on related wild species.

The analyses of the samples collected in this study demonstrated, through genomic sequencing and serological methods, the circulation of a bovine coronavirus in the alpine marmot population. This event is of great interest because it supports a spillover event and the ability of bovine coronavirus to infect rodents. Very little is known about the circulation of coronaviruses in marmot populations. However, surveillance conducted in marmots in the same areas in 2021 revealed the presence of only one coronavirus-positive sample closely related to beta-coronavirus detected in hares, rabbits, and rodents [20]. Instead, this study detected a beta-coronavirus typically found in cattle and wild ruminants, confirming the species jump event. Genome-detection methods identified BCoV in nine animals divided into two groups which, based on progressive identification numbers (246, 249, 250 and 251, and 332, 336, 339, 340, and 341), corresponded to animals from the same capture locations which were probably part of a family nucleus.

Interestingly, the partial RdRp gene sequencing of all positive faecal samples showed the highest identity with BCoVs detected in roe deer in the Alpine mountains in Italy. Marmots share areas and pastures with roe deer and other wild ruminants, which are widely present in the study area, and this may explain an environmental transmission from ruminants to marmots. Marmots were captured in individual cages, and faecal samples were collected directly from the animal, so the contamination of marmot samples with ruminant faeces can be ruled out. 

Due to their high mutation rates and recombination frequencies, coronaviruses can adapt very quickly to changing ecological niches. In the *Embecoviruses* subgenus, this plasticity may facilitate overcoming host barriers to infect other species and genetically adapt to a new host. Indeed, *Embecoviruses* are characterised by different genome structures in the accessory genes coding for ns4.9 and ns4.8 and by the presence of the HE protein. Although the functions of these accessory proteins are not well understood, it has been hypothesised that they are niche-specific and play a role in virus tropism [21,22]. Also, the HE protein may have a role in increasing the host range of *Embecoviruses* by expanding the number and/or types of cells that the virus can infect [23].

The molecular analysis of the S and HE proteins revealed few amino acid changes compared to the B-CoV sequences in both proteins. The S and HE proteins are located on the surface of the viral particle and are involved in binding to host cell receptors for virus entry. BCov HE has a receptor-binding site that specifically binds 9-*O*-acetylated sialic acid (9-*O*-Ac-Sia) and an esterase domain, which has receptor-destroying enzymatic activity that can remove 9-*O*-Ac-Sia from the cell surface [24]. It was observed that the presence in murine coronaviruses of different aa insertions within the R3 and R4 loops of the HE protein is involved in the switching of the receptor from the 9-*O*-Ac-Sia typical of B-CoV to 4-*O*-Ac-Sia [24]. The aa sequences of the marmot HE proteins showed high homology to the B-CoV sequences, and no aa insertions were found in the R3 and R4 loops (Table S3). No information is available on the presence of cellular receptors in marmots; however, the homology of the sequences, including the R3 and R4 loops, with B-CoV might suggest a binding specificity between marmot coronaviruses and 9-*O*-Ac-Sia. However, further studies are needed to better understand the cellular receptors present in marmots.

Using two serological methods, BCoV infection in marmots was confirmed by detecting specific antibodies in marmot sera. The first test used was a BCoV MAb-based competitive ELISA capable of testing sera from different animal species but only validated for sera from ruminants. It was, therefore, necessary to use the VNT test as a gold standard method to confirm the serological results obtained. VNT titres between 1/10 and 1/80 were detected in all but one of the sera. This serum showed a positivity in ELISA close to the cut-off value not detected in VNT. Unfortunately, the random collection of marmot samples made obtaining faeces and blood samples from the same animals in the two moments of capture and release difficult. This would have been useful in identifying the presence of the viral genome and antibodies in the same individuals. However, the short time frame, a few days, between capture and release would have prevented us from detecting antibodies in virologically positive individuals. 

Surveillance studies in wildlife are essential to better investigate the circulation of coronaviruses in different species, with a particular emphasis on detecting spillover events. 

## 5. Conclusions

This study provides molecular and serological evidence of a bovine coronavirus (BCoV) in a population of alpine marmots. Knowledge about the health status of these populations in the Alps is very limited. Thanks to these results, it was possible to highlight the plasticity of this virus, which has already been shown to pass into different species, although it is generally limited to ruminants, with sporadic identifications in dogs [4]. On the other hand, the marmot could also be a particularly susceptible species. Previous studies in the same area have isolated a coronavirus with 97% identity with a lagomorph coronavirus strain [20]. Such an aspect should be further investigated, considering that this animal lives in sympatry with other domestic and wild species and could, therefore, not only act as a reservoir for specific pathogens, even potentially zoonotic ones, but also facilitate their interspecific transmission.

## Figures and Tables

**Table 1:** Results of blast protein analysis. For each viral protein, the highest percentage of identity, the most closely related strain, and its GenBank accession number are shown.

Protein	% of Identity	Strain Name, Country, Year of Isolation	GenBank Accession Number
1ab	99.46	Bovine/ICSA16-EN, France, 2014	MG757139
1a	99.43	Bovine/ICSA16-EN, France, 2014	MG757139
32KDA	99.51	Buffalo, Italy/179/07-11, Italy, 2007	EU019216
HE	99.53	Bovine/S3, Ireland, 2022	OR271241
S	98.61	Bovine/RSVPIV3-221026, Ireland, 2022	PP156987
4.9KDA	96.55	Bovine, B298, China, 2021	OP866728
4.8KDA	97.62	Capra hircus, CH-4-22-01, USA, 2022	OP004056
E	100	Bovine-ENT, USA	NC003045
M	99.13	Bovine, LSV-94LSS-051, USA	P69599
N	99.33	Bovine, EPI/Caen/2008/04, France, 2008	KT318086
I	97.75	Bovine, IND/2021/N77, India, 2021	OP820539

**Table 2:** Results of serological analyses. Positive samples are highlighted in grey.

ID	Protocol Number	BCoV Ab ELISA > 75%	BCoV VNT ≥1/10
166	179,405/1	93	1/40
167	179,405/2	94	1/80
168	179,405/3	7	NEG
169	179,405/4	94	1/80
170	179,405/5	92	1/20
171	179,405/6	93	1/40
172	179,405/7	94	1/80
174	179,405/8	13	NEG
176	179,405/9	70	NEG
181	179,405/10	30	NEG
182	179,405/11	30	NEG
183	179,405/12	9	NEG
184	179,405/13	11	NEG
185	179,405/14	75	NEG
186	179,405/15	25	NEG
213	179,405/16	22	NEG
214	179,405/17	94	1/10
215	179,405/18	51	NEG
216	179,405/19	75	NEG
217	179,405/20	53	NEG
218	179,405/21	23	NEG
219	179,405/22	14	NEG
220	179,405/23	39	NEG
221	179,405/24	18	NEG
222	179,405/25	38	NEG
303	179,405/26	58	NEG
306	179,405/27	81	NEG
307	179,405/28	71	NEG
308	179,405/29	36	NEG
309	179,405/30	33	NEG
310	179,405/31	64	NEG
311	179,405/32	30	NEG
312	179,405/33	45	NEG
313	179,405/34	53	NEG
315	179,405/35	37	NEG

## Data Availability

All data are reported in the main text.

## Supplementary Materials

The following supporting information can be downloaded at: https://www.mdpi.com/article/10.3390/v16040591/s1, Table S1. Areas and dates of capture and release of marmots and the sex, age, body measurements, and samples collected from each animal (Y = yes; N = not collected). Table S2. Molecular analysis of the amino acid (aa) sequences of the complete S protein of Italian marmots and reference and field B-CoVs. Immune reactive domains (red) and regions important for virus–cell interactions (blue) are shown in boxes. Particular aa changes suggested as being linked to virulence factors (red), and enteric (green) and respiratory (blue) tropism are indicated by arrows [25]. Amino acid changes observed only in the marmot sequences are evidenced in yellow. Table S3. Molecular analysis of the amino acid sequences (aa) of the complete HE protein of Italian marmots and reference and field B-CoVs. The domains of the HE protein, the R3 and R4 loops, and the receptor-binding domain (RBD) are highlighted in boxes [26]. Amino acid changes observed only in the marmot sequences are highlighted in yellow.
