# Supplementary material for: Molecular and Serological Detection of Bovine Coronaviruses in Marmots (Marmota marmota) in the Alpine Region
Source: Viruses. 2024 Apr 11;16(4):591. doi: 10.3390/v16040591 (PMC11054042; doi:10.3390/v16040591)
Supplement: Supplementary file 1 [file viruses-16-00591-s001.zip › viruses-2934756-supplementary.pdf]

ID	Capture date	Capture area		Release date	Release area		Sex	Age	Body weight	Body length	Samples		Pan-CoV RT-PCR
		Area	Prov		Area	Prov		Adult (Ad) Juvenile (Juv)	Kg	Cm	Faeces	Sera	
159	08/05/2022	Viera	So	12/05/2022	Tombea, Magasa	Bs	M	Ad	3.25	48.0	Y	N	NEG
160	08/05/2022	Viera	So	12/05/2022	Tombea, Magasa	Bs	M	Ad	3.63	45.0	Y	N	NEG
161	08/05/2022	Viera	So	12/05/2022	Tombea, Magasa	Bs	M	Juv	2.42	41.0	Y	N	NEG
162	08/05/2022	Viera	So	12/05/2022	Tombea, Magasa	Bs	F	Ad	2.58	39.0	Y	N	NEG
163	08/05/2022	Viera	So	12/05/2022	Tombea, Magasa	Bs	F	Ad	3.57	45.5	Y	N	NEG
165	07/05/2022	Federia	So	12/05/2022	Tremalzo, Tremosine	Bs	M	Juv	1.55	36.5	Y	N	NEG
166	07/05/2022	Federia	So	12/05/2022	Tremalzo, Tremosine	Bs	F	Ad	3.02	44.0	Y	Y	NEG
167	09/05/2022	Viera	So	12/05/2022	Tombea, Magasa	Bs	F	Ad	3.90	49.0	Y	Y	NEG
168	09/05/2022	Viera	So	12/05/2022	Tombea, Magasa	Bs	M	Ad	3.75	48.0	Y	Y	NEG
169	09/05/2022	Viera	So	12/05/2022	Tombea, Magasa	Bs	F	Juv	2.11	40.5	N	Y	NEG
170	09/05/2022	Viera	So	12/05/2022	Tombea, Magasa	Bs	F	Ad	3.48	48.3	Y	Y	NEG
171	09/05/2022	Viera	So	12/05/2022	Tombea, Magasa	Bs	M	Ad	3.61	49.5	Y	Y	NEG
172	09/05/2022	Viera	So	12/05/2022	Tombea, Magasa	Bs	F	Ad	4.04	49.5	N	Y	NEG
174	09/05/2022	Viera	So	12/05/2022	Tombea, Magasa	Bs	F	Ad	3.49	48.5	Y	Y	NEG
175	09/05/2022	Viera	So	12/05/2022	Tombea, Magasa	Bs	M	Ad	3.52	51.0	Y	N	NEG
176	09/05/2022	Viera	So	12/05/2022	Tombea, Magasa	Bs	F	Ad	3.30	44.5	Y	Y	NEG
177	10/05/2022	Viera	So	12/05/2022	Tombea, Magasa	Bs	M	Ad	4.21	48.2	Y	N	NEG
181	10/05/2022	Viera	So	12/05/2022	Tombea, Magasa	Bs	F	Ad	3.51	43.0	Y	Y	NEG
182	10/05/2022	Viera	So	12/05/2022	Tombea, Magasa	Bs	M	Juv	1.60	37.0	N	Y	NEG
183	10/05/2022	Federia_alta	So	12/05/2022	Tremalzo, Tremosine	Bs	M	Juv	2.49	38.5	N	Y	NEG
184	10/05/2022	Federia_alta	So	12/05/2022	Tremalzo, Tremosine	Bs	M	Juv	2.48	40.5	N	Y	NEG
185	10/05/2022	Federia_alta	So	12/05/2022	Tremalzo, Tremosine	Bs	F	Ad	4.27	46.0	N	Y	NEG
186	10/05/2022	Federia_alta	So	12/05/2022	Tremalzo, Tremosine	Bs	M	Ad	4.21	48.5	N	Y	NEG
205	13/05/2022	Federia_media	So	18/05/2022	Aviolo	Bs	M	Ad	3.94	50.0	Y	N	NEG
206	13/05/2022	Federia_media	So	16/05/2022	Tombea, Magasa	Bs	M	Ad	2.90	43.5	Y	N	NEG
207	13/05/2022	Federia_media	So	16/05/2022	Tombea, Magasa	Bs	M	Ad	3.43	45.0	Y	N	NEG
208	13/05/2022	Federia_media	So	16/05/2022	Tombea, Magasa	Bs	M	Ad	3.71	47.5	Y	N	NEG
209	13/05/2022	Federia_media	So	16/05/2022	Tombea, Magasa	Bs	M	Juv	2.01	41.5	Y	N	NEG
210	13/05/2022	Federia_media	So	16/05/2022	Tombea, Magasa	Bs	M	Ad	3.57	49.5	Y	N	NEG
211	13/05/2022	Federia_media	So	16/05/2022	Tombea, Magasa	Bs	M	Ad	3.97	47.0	Y	N	NEG
212	13/05/2022	Federia_media	So	18/05/2022	Aviolo	Bs	M	Ad	3.34	46.5	Y	N	NEG
213	13/05/2022	Federia_media	So	16/05/2022	Tombea, Magasa	Bs	M	Ad	3.62	49.5	N	Y	NEG
214	13/05/2022	Federia_media	So	16/05/2022	Tombea, Magasa	Bs	M	Ad	3.18	45.0	N	Y	NEG
215	13/05/2022	Federia_media	So	16/05/2022	Tombea, Magasa	Bs	M	Ad	3.58	48.0	N	Y	NEG
216	13/05/2022	Federia_media	So	16/05/2022	Tombea, Magasa	Bs	M	Ad	3.93	50.5	Y	Y	NEG
217	13/05/2022	Federia_media	So	16/05/2022	Tombea, Magasa	Bs	M	Ad	4.12	49.0	Y	Y	NEG
218	13/05/2022	Federia_media	So	18/05/2022	Aviolo	Bs	M	Ad	3.87	49.5	Y	Y	NEG
219	14/05/2022	Federia_media	So	16/05/2022	Tombea, Magasa	Bs	M	Ad	2.95	47.0	N	Y	NEG
220	14/05/2022	Vallaccia	So	18/05/2022	Aviolo	Bs	M	Ad	3.51	49.0	Y	Y	NEG
221	14/05/2022	Vallaccia	So	18/05/2022	Aviolo	Bs	M	Juv	1.90	48.5	N	Y	NEG
222	14/05/2022	Vallaccia	So	18/05/2022	Aviolo	Bs	M	Juv	1.75	47.0	N	Y	NEG

223	15/05/2022	Vallaccia	So	18/05/2022	Aviolo	Bs	M	Ad	3.23	48.0	Y	N	NEG
224	15/05/2022	Vallaccia	So	18/05/2022	Aviolo	Bs	M	Ad	3.74	51.5	Y	N	NEG
226	16/05/2022	Vallaccia	So	18/05/2022	Aviolo	Bs	M	Ad	3.89	51.0	Y	N	NEG
228	16/05/2022	Federia_bassa	So	18/05/2022	Aviolo	Bs	M	Ad	3.59	50.0	Y	N	NEG
230	16/05/2022	Federia_bassa	So	18/05/2022	Aviolo	Bs	M	Ad	3.94	50.0	Y	N	NEG
231	16/05/2022	Federia_bassa	So	18/05/2022	Aviolo	Bs	M	Ad	3.89	50.0	Y	N	NEG
234	16/05/2022	Federia_bassa	So	18/05/2022	Aviolo	Bs	M	Ad	3.92	50.0	Y	N	NEG
236	16/05/2022	Vallaccia	So	18/05/2022	Aviolo	Bs	M	Ad	3.80	49.0	Y	N	NEG
238	17/05/2022	Vallaccia	So	18/05/2022	Aviolo	Bs	M	Juv	2.24	40.0	Y	N	NEG
241	18/05/2022	Calcheira_Superiore	So	22/05/2022	Aviolo	Bs	M	Ad	3.98	51.0	Y	N	NEG
242	18/05/2022	Bork	So	22/05/2022	Aviolo	Bs	M	Ad	4.13	50.0	Y	N	NEG
243	19/05/2022	Bork	So	22/05/2022	Aviolo	Bs	M	Ad	2.83	42.0	Y	N	NEG
244	19/05/2022	Bork	So	22/05/2022	Aviolo	Bs	M	Ad	3.93	49.0	Y	N	NEG
246	19/05/2022	Bork	So	22/05/2022	Aviolo	Bs	M	Ad	4.44	50.0	Y	N	POS
249	20/05/2022	Bork	So	22/05/2022	Aviolo	Bs	M	Ad	2.68	44.5	Y	N	POS
250	20/05/2022	Bork	So	22/05/2022	Aviolo	Bs	M	Ad	3.81	49.5	Y	N	POS
251	20/05/2022	Bork	So	22/05/2022	Aviolo	Bs	M	Ad	2.9	43.5	Y	N	POS
252	21/05/2022	Beltram	So	22/05/2022	Aviolo	Bs	M	Ad	4.62	49.5	Y	N	NEG
253	21/05/2022	Bork	So	22/05/2022	Aviolo	Bs	M	Ad	4.1	51.5	Y	N	NEG
255	21/05/2022	Bork	So	22/05/2022	Aviolo	Bs	M	Juv	2.3	43.5	Y	N	NEG
302	12/05/2022	Federia_alta	So	16/05/2022	Tombea, Magasa	Bs	F	Ad	2.77	44.0	Y	N	NEG
303	12/05/2022	Federia_media	So	16/05/2022	Tombea, Magasa	Bs	F	Ad	3.29	46.0	Y	Y	NEG
305	12/05/2022	Federia_media	So	18/05/2022	Aviolo	Bs	F	Ad	3.07	41.0	Y	N	NEG
306	13/05/2022	Federia_media	So	16/05/2022	Tombea, Magasa	Bs	F	Ad	3.37	46.5	N	Y	NEG
307	13/05/2022	Federia_media	So	16/05/2022	Tombea, Magasa	Bs	F	Ad	3.78	48.5	Y	Y	NEG
308	13/05/2022	Federia_media	So	16/05/2022	Tombea, Magasa	Bs	F	Juv	1.78	38.5	N	Y	NEG
309	14/05/2022	Federia_media	So	16/05/2022	Tombea, Magasa	Bs	F	Ad	2.74	48.5	N	Y	NEG
310	14/05/2022	Federia_media	So	18/05/2022	Aviolo	Bs	F	Ad	2.97	49.0	Y	Y	NEG
311	14/05/2022	Federia_media	So	18/05/2022	Aviolo	Bs	F	Ad	3.35	49.0	N	Y	NEG
312	14/05/2022	Federia_bassa	So	18/05/2022	Aviolo	Bs	F	Ad	3.31	47.0	Y	Y	NEG
313	14/05/2022	Vallaccia	So	18/05/2022	Aviolo	Bs	F	Ad	3.21	47.0	N	Y	NEG
314	15/05/2022	Federia_bassa	So	18/05/2022	Aviolo	Bs	F	Ad	3.53	47.5	Y	N	NEG
315	15/05/2022	Federia_bassa	So	18/05/2022	Aviolo	Bs	F	Ad	3.29	48.5	N	Y	NEG
316	15/05/2022	Vallaccia	So	18/05/2022	Aviolo	Bs	F	Ad	3.33	50.0	Y	N	NEG
317	16/05/2022	Vallaccia	So	18/05/2022	Aviolo	Bs	F	Ad	3.45	46.0	Y	N	NEG
318	16/05/2022	Federia_bassa	So	18/05/2022	Aviolo	Bs	F	Ad	3.44	48.5	Y	N	NEG
320	16/05/2022	Vallaccia	So	18/05/2022	Aviolo	Bs	F	Ad	3.25	44.0	Y	N	NEG
322	16/05/2022	Campaccia	So	18/05/2022	Aviolo	Bs	F	Juv	1.76	46.5	Y	N	NEG
323	17/05/2022	Vallaccia	So	18/05/2022	Aviolo	Bs	F	Ad	3.52	42.5	Y	N	NEG
327	18/05/2022	Calcheira_Superiore	So	22/05/2022	Aviolo	Bs	F	Ad	3.87	48.0	Y	N	NEG
332	19/05/2022	Bork	So	22/05/2022	Aviolo	Bs	F	Ad	3.43	45.0	Y	N	POS
334	20/05/2022	Bork	So	22/05/2022	Aviolo	Bs	F	Juv	2.47	42.0	Y	N	NEG
335	20/05/2022	Bork	So	22/05/2022	Aviolo	Bs	F	Juv	2.28	41.0	Y	N	NEG
336	20/05/2022	Bork	So	22/05/2022	Aviolo	Bs	F	Juv	2.39	40.5	Y	N	POS
339	20/05/2022	Bork	So	22/05/2022	Aviolo	Bs	F	Ad	4.18	50.5	Y	N	POS
340	20/05/2022	Bork	So	22/05/2022	Aviolo	Bs	F	Ad	3.85	46.5	Y	N	POS

341	20/05/2022	Bork	So	22/05/2022	Aviolo	Bs	F	Ad	3.5	46.5	Y	N	POS
342	21/05/2022	Bork	So	22/05/2022	Aviolo	Bs	F	Juv	2.38	41.5	Y	N	

ID: Identificative number

Prov: Province; So: Sondrio; Bs: Brescia

M: male; F: female

Y:yes; N: not

Table S1. Areas and dates of capture and release of marmots and the sex, age, body measurements, and samples collected from each animal (Y = yes; N = not collected).

S protein

Signal peptide

```
                                                                                10        20        30        40        50        60        70        80        90       100       110       120
#U00735.2Bovine_coronavirus_strain_Mebus_complete_genome                         MFLILLISLP MAFAVIGDLK CTTVSINDVD TGAPSISTDI VDVTNGLGTY YVLDRVYLNT TLLLNGYYPT SGSTYRNMAL KGTLLLSRLW FKPPFLSDFI NGIFAKVKNT KVIKKGVMYS [ 120]
#NC_003045_Bovine_coronavirus_isolate_BCoV-ENTSprotein_USA                       .......... T......... .......... ..V......T .......... .......... .......... .......... .......T.. .........T .......... ....D..... [ 120]
#EF424615.1Bovine_coronavirus_E-AH65_complete_genome                             .......... T......... ...L...... ..V......T .......... .......... .......... .......... .......T.. .........T .......... ....D..... [ 120]
#AF220295.1Bovine_coronavirus_strain_Quebec_complete_genome                      .......... .......... .......... .......... .......... .......... .......... .......... .......... .......... .......... .......... [ 120]
#KT318118.1_Bovine_coronavirus_isolate_BCoV/FRA/EPI/Caen/2013/08_spike_glycoprotein .......... T......... .......... ..V......T .......... .......... .......... .......... .......T.. .........T .......... ....N..... [ 120]
#EU814648.1_Bovine_coronavirus_strain_438/06-TN-50_S_protein_(S)_gene_complete_cds .......... T......... ..SR...... ..V..T..AT .......... .......... .......... .......... .......T.. .........T .......... ..V....... [ 120]
#KF169910.1_Bovine_coronavirus_isolate_SWE/02-2_spike_glycoprotein_(S)_gene_complete_cds .......... T......... .......... ..V.F....T .......... .......... .......... .......... .......T.. .........T .......... ..T.D..... [ 120]
#KF169913.1_Bovine_coronavirus_isolate_DEN/03-1_spike_glycoprotein_(S)_gene_complete_cds .......... T......... .......... ..V......T .......... .......... .......... .......... .......T.. .........T .......... ..T.DR.... [ 120]
#KF169934.1_Bovine_coronavirus_isolate_SWE/P/09-1_spike_glycoprotein_(S)_gene_complete_cds .......... TT........ .......... ..V.F....T .......... .......... .......... .......... .......T.. .........T .......... ....N..... [ 120]
#EU019216_Bovine_coronavirus_Bubalus/Italy/179/07-11gene_spike_protein_(S)_4.9_kDs .......... T......... .......... ..V......T .......... .......... .......... .......... .......T.. .........T .......... ....N..... [ 120]
#DQ389632.1_Bovine_coronavirus_isolate_KCD1_spike_glycoprotein_precursor_(S)_KOR  .......... .......... .......... ..V......T .......... .......... .......... .......... .......T.. .........T .......... ....D..... [ 120]
#DQ389658.1_Bovine_coronavirus_isolate_KWD17_spike_glycoprotein_precursor_(S)_KOR .......... .......... .......... ..V......T .......... .......... .......... .......... .......T.. .........T .......... ....D..... [ 120]
#KF169932.1_Bovine_coronavirus_isolate_SWE/C/08-2_spike_glycoprotein_(S)_gene_complete_cds .......... TT........ .........N ..V.L....T .......... .......... .......... .......... .......T.. .........T .......... ....N..... [ 120]
#KF169916.1_Bovine_coronavirus_isolate_DEN/05-1_spike_glycoprotein_(S)_gene_complete_cds .......... .......... .......... ..V......T .......... .......... .......... .......... .......T.. .......H.T .......... ..T.D..... [ 120]
#KF169918.1_Bovine_coronavirus_isolate_DEN/05-3_spike_glycoprotein_(S)_gene_complete_cds .......... .......... .......... ..V......T .......... .......... .......... .......... .......T.. .......H.T .......... ..T.D..... [ 120]
#EF193075.1_Bovine_coronavirus_strain_V270_1983_GER_spike_protein_gene           .......... .......... .......... .......... .......... .......... .......... .......... .......... .......... .......... .......... [ 120]
#DQ389637.1_Bovine_coronavirus_isolate_KCD6_spike_glycoprotein_precursor_(S)_gene-KOR ......N... T......... .......... ..V......T .......... .......... .......... .......... .......T.. .........T .......... ....G..... [ 120]
#KF169908.1_Bovine_coronavirus_isolate_SWE/C/92_spike_glycoprotein_(S)_gene_complete_cds .......... T......... .......... ..V......T .......... .......... .......... .......... .......T.. .........T .......... ..T.D..... [ 120]
#D00731.1_Bovine_enteric_coronavirus_F15_1979_FRA_gene_for_spike_glycoprotein    .......... ..L....... .......... ..V......T .......... .......... .......... .......... .......T.. .......... .......... ....H..... [ 120]
#ON792942_Bovine_coronavirus_strain_ABGEB0-54_S_gene_Ireland                     .......... T......... .......... ..V......T .......... .......... .......... .......... .......T.. .........T .......... ....N..... [ 120]
#FJ938065.1Bovine_respiratory_coronavirus_AH187_complete_genome                  .......... T......... ...L...... ..V......T .......... .......... .......... .......... .......T.. .........T .......... ....D..... [ 120]
#MG757144.1_Bovine_coronavirus_isolate_strain_ICSA17-LBA_spike_protein_(S)        .......... T......... .......... ..V......T .......... .......... .......... .......... .......T.. .........T .......... ....N..... [ 120]
#MG757140_Bovine_coronavirus_isolate_ICSA16-LBA_2014_France                      .......... T......... .......... ..V......T .......... .......... .......... .......... .......T.. .........T .......... ....N..... [ 120]
#X2127-Marmot/Italy/193728-34/2023/CoV                                           .......... T......... .......... ..V......T .......... .......... .......... .......... .......T.. .........T ....S..... ....N..T.. [ 120]
#X2128-Marmot/Italy/193728-35/2023/CoV                                           .......... T......... .......... ..V......T .......... .......... .......... .......... .......T.. .........T ....S..... ....N..T.. [ 120]

                                                                               130       140       150       160       170       180       190       200       210       220       230       240
#U00735.2Bovine_coronavirus_strain_Mebus_complete_genome                         EFPAITIGST FVNTSYSVVV QPHTTNLDNK LQGLLEISVC QYTMCEYPHT ICHPNLGNKR VELWHWDTGV VSCLYKRNFT YDVNADYLYF HFYQEGGTFY AYFTDTGVVT KFLFNVYLGT [ 240]
#NC_003045_Bovine_coronavirus_isolate_BCoV-ENTSprotein_USA                       .......... .......... .....I.G.. ...F...... ........N. ..N.....Q. .......... .......... .......... .......... .......... .......... [ 240]
#EF424615.1Bovine_coronavirus_E-AH65_complete_genome                             .......... .......... .....I.G.. ...F...... ........N. ..N.....Q. .......... .......... .......... .......... .......... .......... [ 240]
#AF220295.1Bovine_coronavirus_strain_Quebec_complete_genome                      .......... .......... .......... .......... .......... ....K..... .......... .......... .......... .......... .......... .......... [ 240]
#KT318118.1_Bovine_coronavirus_isolate_BCoV/FRA/EPI/Caen/2013/08_spike_glycoprotein .......... .......... ..Y...F... I.....V... ........N. ..N.....Q. .......... .......... .......... .......... .......... .......... [ 240]
#EU814648.1_Bovine_coronavirus_strain_438/06-TN-50_S_protein_(S)_gene_complete_cds .......... .......... .......... .......... ........N. ..N.....R. .......... .......... .......... .......... .......... .......... [ 240]
#KF169910.1_Bovine_coronavirus_isolate_SWE/02-2_spike_glycoprotein_(S)_gene_complete_cds .......... .......... .......... .......... ........N. ..N.....Q. .......... .......... .......... .......... .......F.. .......... [ 240]
#KF169913.1_Bovine_coronavirus_isolate_DEN/03-1_spike_glycoprotein_(S)_gene_complete_cds .......... .......... .......... .......... ........N. ..N.....Q. .......... .......... .......... .......... .......... .......... [ 240]
#KF169934.1_Bovine_coronavirus_isolate_SWE/P/09-1_spike_glycoprotein_(S)_gene_complete_cds .......... .......... ......F... I.....V... ........N. ..N.....Q. .......... .......... .......... .......... .......... .......... [ 240]
#EU019216_Bovine_coronavirus_Bubalus/Italy/179/07-11gene_spike_protein_(S)_4.9_kDs .......... .......... ..Y...F... I.....V... ........N. ..N.....Q. .......... .......... .......... .......... .......... .......... [ 240]
#DQ389632.1_Bovine_coronavirus_isolate_KCD1_spike_glycoprotein_precursor_(S)_KOR  .......... .......... ......F.S. .......... ........N. ........Q. .......... .......... .......... .......... .......... .......... [ 240]
#DQ389658.1_Bovine_coronavirus_isolate_KWD17_spike_glycoprotein_precursor_(S)_KOR .......... .......... ......F.S. .......... ........N. ..N.....Q. .......... .......... .......... .......... .......... .......... [ 240]
#KF169932.1_Bovine_coronavirus_isolate_SWE/C/08-2_spike_glycoprotein_(S)_gene_complete_cds .......... .......... ......F... I.....V... ........N. ..N.....Q. .......... .......... .......... .......... .......... .......... [ 240]
#KF169916.1_Bovine_coronavirus_isolate_DEN/05-1_spike_glycoprotein_(S)_gene_complete_cds ........T. .......... .......... .......... ........N. ..N....... .......... .......... .......... ....K..... .......... .......... [ 240]
#KF169918.1_Bovine_coronavirus_isolate_DEN/05-3_spike_glycoprotein_(S)_gene_complete_cds ........T. .......... .......... .......... ........N. ..N....... .......... .......... .......... ....K..... .......... .......... [ 240]
#EF193075.1_Bovine_coronavirus_strain_V270_1983_GER_spike_protein_gene           .......... .......... .......... .......... .......... ........R. .......... .......... .......... .......... .......... .......... [ 240]
#DQ389637.1_Bovine_coronavirus_isolate_KCD6_spike_glycoprotein_precursor_(S)_gene-KOR .......... .......... ......F.S. .......... ........N. ..N.....Q. .......... .......... .......... .......... .......... .......... [ 240]
#KF169908.1_Bovine_coronavirus_isolate_SWE/C/92_spike_glycoprotein_(S)_gene_complete_cds .......... .L........ .......... .......... ........N. ........Q. .......... .......... .......... .......... .......... .......... [ 240]
#D00731.1_Bovine_enteric_coronavirus_F15_1979_FRA_gene_for_spike_glycoprotein    .......... .......... .......... .......... ........N. ........R. .......... .......... .......... .......... .......... .......... [ 240]
#ON792942_Bovine_coronavirus_strain_ABGEB0-54_S_gene_Ireland                     .......... .......... ......F... I.....V... ........N. ..N.....Q. .......... .......... .......... .......... .......... .......... [ 240]
#FJ938065.1Bovine_respiratory_coronavirus_AH187_complete_genome                  .......... .......... .....I.G.. ...F...... ........N. ..N.....Q. .......... .......... .......... .......... .......... .......... [ 240]
#MG757144.1_Bovine_coronavirus_isolate_strain_ICSA17-LBA_spike_protein_(S)        .......... .......... ......F... I.....V... ........N. ..N.....Q. .......... .......... .......... .......... .......... .......... [ 240]
#MG757140_Bovine_coronavirus_isolate_ICSA16-LBA_2014_France                      .......... .......... ......F... I.....V... ........N. ..N.....Q. .......... .......... .......... .......... .......... .......... [ 240]
#X2127-Marmot/Italy/193728-34/2023/CoV                                           .......... .......... ....I.F... I.....V... ........N. ..N.....Q. .......... .......... .......... .......... .......... .......... [ 240]
#X2128-Marmot/Italy/193728-35/2023/CoV                                           .......... .......... ....I.F... I.....V... ........N. ..N.....Q. .......... .......... .......... .......... .......... .......... [ 240]
```

Immuno-reactive domain SA1

```
                                                                                 250       260       270       280       290       300       310       320       330       340       350       360
#U00735.2Bovine_coronavirus_strain_Mebus_complete_genome                         VLSHYYVLPL TCSSAMTLEY WVTPLTSKQY LLAFNQDGVI FNAVDCKSDF MSEIKCKTLS IAPSTGVYEL NGYTVQPIAD VYRRIPNLPD CNIEAWLNDK SVPSPLNWER KTFSNCNFNM  360]
#NC_003045_Bovine_coronavirus_isolate_BCoV-ENTSprotein_USA                       .......M.. ..N..L.... .......... .......... .......... .......... .......... .......... .......... .......... .......... ..........  360]
#EF424615.1Bovine_coronavirus_E-AH65_complete_genome                             .......M.. ..N..L.... .......... .......... .......... .......... .......... .......... .......... .......... .......... ..........  360]
#AF220295.1Bovine_coronavirus_strain_Quebec_complete_genome                      .......... .......... .......... .......... .......... .......... .......... .......... .......... .......... .......... ..........  360]
#KT318118.1_Bovine_coronavirus_isolate_BCoV/FRA/EPI/Caen/2013/08_spike_glycoprotein .......M.. ..N..LN... .......... .......... .......... .......... .......... .......... .......... .......... .......... ..........  360]
#EU814648.1_Bovine_coronavirus_strain_438/06-TN-50_S_protein_(S)_gene_complete_cds .......M.. ..N..LN... .......... .......... .......... .......... .......... .......... .......... .......... .......... ..........  360]
#KF169910.1_Bovine_coronavirus_isolate_SWE/02-2_spike_glycoprotein_(S)_gene_complete_cds .......M.. ..N..LN... .......... .......... .......... .......... .......... .......... .......... .......... .......... ..........  360]
#KF169913.1_Bovine_coronavirus_isolate_DEN/03-1_spike_glycoprotein_(S)_gene_complete_cds .......M.. ..N..LN... .......... .......... .......... .......... ...H...... .......... .......... .......... .......... ..........  360]
#KF169934.1_Bovine_coronavirus_isolate_SWE/P/09-1_spike_glycoprotein_(S)_gene_complete_cds .......M.. ..N..LK... .......... .......... .......... .......... .......... .......... .......... .......... .......... ..........  360]
#EU019216_Bovine_coronavirus_Bubalus/Italy/179/07-11gene_spike_protein_(S)_4.9_kDs .......M.. ..N..LN... .......... .......... .......... .......... .......... .......T.. .......F.. .......... .......... ..........  360]
#DQ389632.1_Bovine_coronavirus_isolate_KCD1_spike_glycoprotein_precursor_(S)_KOR  .......M.. ..N..L.... .......... .......... .......... .......... .......... .......... .......... .......... .......... ..........  360]
#DQ389658.1_Bovine_coronavirus_isolate_KWD17_spike_glycoprotein_precursor_(S)_KOR ..L....M.. ..N..L.... .......... .......... .......... .......... .......... .......... .......... .......... .......... ..........  360]
#KF169932.1_Bovine_coronavirus_isolate_SWE/C/08-2_spike_glycoprotein_(S)_gene_complete_cds L......M.. ..N..LN... .......... .......... .......... .......... .......... .......... .......... .......... .......... ..........  360]
#KF169916.1_Bovine_coronavirus_isolate_DEN/05-1_spike_glycoprotein_(S)_gene_complete_cds .......M.. ..N..LN... .......... .......... .......... .......... ...A...... .......... .......... .......... .......... ..........  360]
#KF169918.1_Bovine_coronavirus_isolate_DEN/05-3_spike_glycoprotein_(S)_gene_complete_cds .......M.. ..N..LN... .......... .......... .......... .......... ...A...... .......... .......... .......... .......... ..........  360]
#EF193075.1_Bovine_coronavirus_strain_V270_1983_GER_spike_protein_gene           .......... .......... .......... .......... .......... .......... .......... .......... .......... .......... .......... ..........  360]
#DQ389637.1_Bovine_coronavirus_isolate_KCD6_spike_glycoprotein_precursor_(S)_gene-KOR .......M.. ..N..L.... .......... .......... .......... .......... .......... S......... .......F.. .......... .......... ..........  360]
#KF169908.1_Bovine_coronavirus_isolate_SWE/C/92_spike_glycoprotein_(S)_gene_complete_cds .......M.. ..N..L.... .......... .......... ......N... .......... .......... .......... .......... .......... .......... ..........  360]
#D00731.1_Bovine_enteric_coronavirus_F15_1979_FRA_gene_for_spike_glycoprotein    .......M.. ..N....... .......... .......... .......... .......... .......... .......... .......... .......... .......... ..........  360]
#ON792942_Bovine_coronavirus_strain_ABGEB0-54_S_gene_Ireland                     .......M.. ..N..L.... .......... .......... .......... .......... .......... .......... .......... .......... .......... ..........  360]
#FJ938065.1Bovine_respiratory_coronavirus_AH187_complete_genome                  .......M.. ..N..L.... .......... .......... .......... .......... .......... .......... .......... .......... .......... ..........  360]
#MG757144.1_Bovine_coronavirus_isolate_strain_ICSA17-LBA_spike_protein_(S)        .......M.. ..N..L.... .......... .......... .......... .......... .......... .......... .......... .......... .......... ..........  360]
#MG757140_Bovine_coronavirus_isolate_ICSA16-LBA_2014_France                      .......M.. ..N..L.... .......... .......... .......... .......... .......... .......... .......... .......... .......... ..........  360]
```

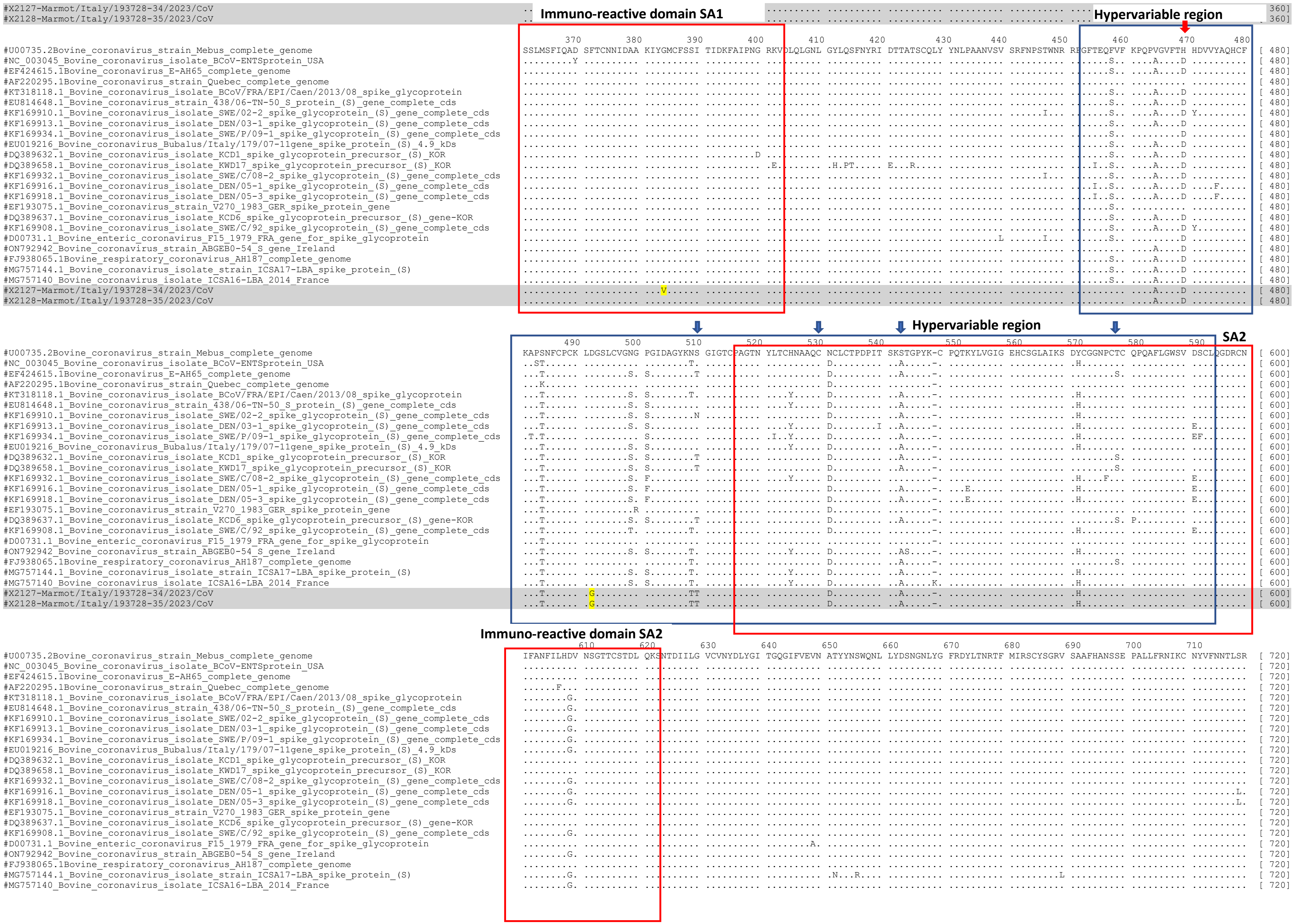
Immuno-reactive domain SA1
Hypervariable region
Hypervariable region
SA2
Immuno-reactive domain SA2

```
#X2127-Marmot/Italy/193728-34/2023/CoV                                                    ........G. .......... .......... .......... .......... .......... .......... .......... .......... .......... .......... ..........  [ 720]
#X2128-Marmot/Italy/193728-35/2023/CoV                                                    ........G. .......... .......... .......... .......... .......... .......... .......... .......... .......... .......... ..........  [ 720]
```

Proteolytic cleavage region

```
                                                                                                 730        740        750        760        770        780        790        800        810        820        830
#U00735.2Bovine_coronavirus_strain_Mebus_complete_genome                                  QLQPINYFDS YLGCVVNADN STSSVVQTCD LTVGSGYCVD YSTKRRSRRA ITTGYRFTTF EPFTVNSVND SLEPVGGLYE IQIPSEFTIG NMEEFIQTSS PKVTIDCSAF VCGDYAACKS  [ 840]
#NC_003045_Bovine_coronavirus_isolate_BCoV-ENTSprotein_USA                                .......... .......... .......... .......... .........S ........N. .......... .......... .......... .......... .......... ..........  [ 840]
#EF424615.1Bovine_coronavirus_E-AH65_complete_genome                                      .......... .......... .......... .......... .........S ........N. .......... .......... .......... .......... .......... ..........  [ 840]
#AF220295.1Bovine_coronavirus_strain_Quebec_complete_genome                               .......... .......... .......... .......... .......... ........N. .......... .......... .......... .......... .......... ..........  [ 840]
#KT318118.1_Bovine_coronavirus_isolate_BCoV/FRA/EPI/Caen/2013/08_spike_glycoprotein       .......... .......... .......... .......... .......... ........N. .....K.... .......... .....D.... .......... ........T. ..........  [ 840]
#EU814648.1_Bovine_coronavirus_strain_438/06-TN-50_S_protein_(S)_gene_complete_cds        .......... .......... .......... .......... .......... ........N. .....K.... .......... .....D.... .......... ........T. ..........  [ 840]
#KF169910.1_Bovine_coronavirus_isolate_SWE/02-2_spike_glycoprotein_(S)_gene_complete_cds  ... ....... .......... .......... .......... .......... ........N. .......... .......... .......... .......... .......... ..........  [ 840]
#KF169913.1_Bovine_coronavirus_isolate_DEN/03-1_spike_glycoprotein_(S)_gene_complete_cds  ..R....... .......... .......... .......... .......... ........N. .......... .......... .......... .......... .......... ..........  [ 840]
#KF169934.1_Bovine_coronavirus_isolate_SWE/P/09-1_spike_glycoprotein_(S)_gene_complete_cds .......... .......... .......... .......... .......... ........N. .......... .......... .......... .......... .......... ..........  [ 840]
#EU019216_Bovine_coronavirus_Bubalus/Italy/179/07-11gene_spike_protein_(S)_4.9_kDs        .......... .......... .......... .......... .........S ........N. .....K.... .......... .....D.... .......... ........T. ..........  [ 840]
#DQ389632.1_Bovine_coronavirus_isolate_KCD1_spike_glycoprotein_precursor_(S)_KOR          .......V.. .......... .......... .......... .........S ........N. .......... .......... .......... .......... .......... ..........  [ 840]
#DQ389658.1_Bovine_coronavirus_isolate_KWD17_spike_glycoprotein_precursor_(S)_KOR         .......... .......... ..RC.KP... .......... .........S ........N. .......... .......... .......... .......... .......... ..........  [ 840]
#KF169932.1_Bovine_coronavirus_isolate_SWE/C/08-2_spike_glycoprotein_(S)_gene_complete_cds .......... .......... .......... .......... .......... ........N. .......... .......... .......... .......... .......... ..........  [ 840]
#KF169916.1_Bovine_coronavirus_isolate_DEN/05-1_spike_glycoprotein_(S)_gene_complete_cds  .......... .......... .......... .......... .......... ........N. .......... .......... .......... .......... .......... ..........  [ 840]
#KF169918.1_Bovine_coronavirus_isolate_DEN/05-3_spike_glycoprotein_(S)_gene_complete_cds  .......... .......... .......... .......... .......... ........N. .......... .......... .......... .......... .......... ..........  [ 840]
#EF193075.1_Bovine_coronavirus_strain_V270_1983_GER_spike_protein_gene                    .......... .......... .......... .......... .......... ........N. .......... .......... .......... .......... .......... ..........  [ 840]
#DQ389637.1_Bovine_coronavirus_isolate_KCD6_spike_glycoprotein_precursor_(S)_gene-KOR     .......... .......... .......... .......... .........S ........N. .......... .......... .......... .......... .......... ..........  [ 840]
#KF169908.1_Bovine_coronavirus_isolate_SWE/C/92_spike_glycoprotein_(S)_gene_complete_cds  .......... .......... .......... .......... .........S ........N. .......... .......... .......... .......... .......... ..........  [ 840]
#D00731.1_Bovine_enteric_coronavirus_F15_1979_FRA_gene_for_spike_glycoprotein             .......... .......... ..A.A..... .......... ........V. ........N. .......... .......... .......... .......... .......... ....C.....  [ 840]
#ON792942_Bovine_coronavirus_strain_ABGEB0-54_S_gene_Ireland                              H......... .......... .......... .......... .......... ........N. .....K.... .......... .....D.... .......... ........T. ..........  [ 840]
#FJ938065.1Bovine_respiratory_coronavirus_AH187_complete_genome                           .......... .......... ...I...... ....N..... .........S ........N. .......... .......... .......... .......... .......... ..........  [ 840]
#MG757144.1_Bovine_coronavirus_isolate_strain_ICSA17-LBA_spike_protein_(S)                .......... .......... .......... .......... .......... ........N. .....K.... .......... .....D.... .......... ........T. ..........  [ 840]
#MG757140_Bovine_coronavirus_isolate_ICSA16-LBA_2014_France                               .......... .......... .......... .......... .......... ........N. .....K.... .......... .....D.... .......... ........T. ..........  [ 840]
#X2127-Marmot/Italy/193728-34/2023/CoV                                                    .M........ .......... .......... .......... .........S ......L.N. .......... .......... .......... .......... .......... ..........  [ 840]
#X2128-Marmot/Italy/193728-35/2023/CoV                                                    .M........ .......... .......... .......... .........S ......L.N. .......... .......... .......... .......... .......... ..........  [ 840]
```

1st hydrophobic region

```
                                                                                                 850        860        870        880        890        900        910        920        930        940        950
#U00735.2Bovine_coronavirus_strain_Mebus_complete_genome                                  QLVEYGSFCD NINAILTEVN ELLDTTQLQV ANSLMNGVTL STKLKDGVNF NVDDINFSPV LGCLGSDCNK VSSRSAIEDL LFSKVKLSDV GFVEAYNNCT GGAEIRDLIC VQSYNGIKVL  [ 960]
#NC_003045_Bovine_coronavirus_isolate_BCoV-ENTSprotein_USA                                .......... .......... .......... .......... .......... .......... .......... .......... .......... .......... .......... ..........  [ 960]
#EF424615.1Bovine_coronavirus_E-AH65_complete_genome                                      .......... .......... .......... .......... .......... .......... .......... .......... .......... .......... .......... ..........  [ 960]
#AF220295.1Bovine_coronavirus_strain_Quebec_complete_genome                               .......... .......... .......... .......... .......... .......... ......A... .......... .......... .......... .......... ..........  [ 960]
#KT318118.1_Bovine_coronavirus_isolate_BCoV/FRA/EPI/Caen/2013/08_spike_glycoprotein       .......... .......... .......... .......... ........K. .......... ...V.....R .......... ......A... .......... .......... ..........  [ 960]
#EU814648.1_Bovine_coronavirus_strain_438/06-TN-50_S_protein_(S)_gene_complete_cds        .......... .......... .......... .......... ........K. .......... ......A..R .......... ......A... .......... .......... ..........  [ 960]
#KF169910.1_Bovine_coronavirus_isolate_SWE/02-2_spike_glycoprotein_(S)_gene_complete_cds  .......... .......... .......... .......... .......... .......... .........R .......... ......A... .......... .......... ..........  [ 960]
#KF169913.1_Bovine_coronavirus_isolate_DEN/03-1_spike_glycoprotein_(S)_gene_complete_cds  .......... .......... .......... .......... .......... .......... .........R .......... ......A... .......... .......... ..........  [ 960]
#KF169934.1_Bovine_coronavirus_isolate_SWE/P/09-1_spike_glycoprotein_(S)_gene_complete_cds .......... .......... .......... .......... .......... .......... .........R .......... ......A... .......... .......... ..........  [ 960]
#EU019216_Bovine_coronavirus_Bubalus/Italy/179/07-11gene_spike_protein_(S)_4.9_kDs        .......... .......... .......... .......... ........K. .......... .........R .......... ......A... .......... .......... ..........  [ 960]
#DQ389632.1_Bovine_coronavirus_isolate_KCD1_spike_glycoprotein_precursor_(S)_KOR          .......... .......... .......... .......... .......... .......... .......... .......... .......... .......... .......... ..........  [ 960]
#DQ389658.1_Bovine_coronavirus_isolate_KWD17_spike_glycoprotein_precursor_(S)_KOR         .......... .......... .......... .......... .......... .......... .......... .......... .......... ..F....... .......... ..........  [ 960]
#KF169932.1_Bovine_coronavirus_isolate_SWE/C/08-2_spike_glycoprotein_(S)_gene_complete_cds .......... .......... .......... .....K.... .......... .......... .........R .......... ......A... .......... .......... ..........  [ 960]
#KF169916.1_Bovine_coronavirus_isolate_DEN/05-1_spike_glycoprotein_(S)_gene_complete_cds  .......... .......... .......... .......... .......... .......... .........R .......... ......A... .......... .......... ..........  [ 960]
#KF169918.1_Bovine_coronavirus_isolate_DEN/05-3_spike_glycoprotein_(S)_gene_complete_cds  .......... .......... .......... .......... .......... .......... .........R .......... ......A... .......... .......... ..........  [ 960]
#EF193075.1_Bovine_coronavirus_strain_V270_1983_GER_spike_protein_gene                    .......... .......... .......... .......... .......... .......... ......Y... .......... .......... .......... .......... ..........  [ 960]
#DQ389637.1_Bovine_coronavirus_isolate_KCD6_spike_glycoprotein_precursor_(S)_gene-KOR     .......... .......... .......... .......... .......... .......... .......... .......... .......... .......... .......... ..........  [ 960]
#KF169908.1_Bovine_coronavirus_isolate_SWE/C/92_spike_glycoprotein_(S)_gene_complete_cds  .......... .......... .......... .......... .......... .......... ..W......R .......... ......A... .......... .......... ..........  [ 960]
#D00731.1_Bovine_enteric_coronavirus_F15_1979_FRA_gene_for_spike_glycoprotein             .......... .......... .......... .......... .......... .......... .....E.... .......... .......... .......... .......... ..........  [ 960]
#ON792942_Bovine_coronavirus_strain_ABGEB0-54_S_gene_Ireland                              .......... .......... .......... .......... ........K. .......... ...V.....R .......... ......A... .......... .......... ..........  [ 960]
#FJ938065.1Bovine_respiratory_coronavirus_AH187_complete_genome                           .......... .......... .......... .......... .......... .......... .......... .......... .......... .......... .......... ..........  [ 960]
#MG757144.1_Bovine_coronavirus_isolate_strain_ICSA17-LBA_spike_protein_(S)                .......... .......... .......... .......... ........K. .......... ...V.....R .......... ......A... .......... .......... ..........  [ 960]
#MG757140_Bovine_coronavirus_isolate_ICSA16-LBA_2014_France                               .......... .......... .......... .......... ........K. .......... ...V.....R .......... ......A... .......... .......... ..........  [ 960]
#X2127-Marmot/Italy/193728-34/2023/CoV                                                    .......... .......... .......... .......... .......... .......... .......... .......... .......... .......... .......... ..........  [ 960]
#X2128-Marmot/Italy/193728-35/2023/CoV                                                    .......... .......... .......... .......... .......... .......... .......... .......... .......... .......... .......... ..........  [ 960]
```

1st hydrophobic region

HR-N domain

```
                                                                                                 970        980        990       1000       1010       1020       1030       1040       1050       1060       1070
#U00735.2Bovine_coronavirus_strain_Mebus_complete_genome                                  PPLLSVNQIS GYTLAATSAS LFPPLSAAVG VPFYLNVQYR INGIGVTMDV LSQNQKLIAN AFNNALDAIQ EGFDATNSAL VKIQAVVNAN AEALNNLLQQ LSNRFGAISS SLQEILSRLD  [1080]
#NC_003045_Bovine_coronavirus_isolate_BCoV-ENTSprotein_USA                                .....E.... .......... ....W...A. .......... .......... .......... ......G... .......... .......... .......... .......... ..........  [1080]
#EF424615.1Bovine_coronavirus_E-AH65_complete_genome                                      .....E.... .......... ....W...A. .......... .......... .......... ......G... .......... .......... .......... .......... ..........  [1080]
#AF220295.1Bovine_coronavirus_strain_Quebec_complete_genome                               .......... .......... .......... .......... .......... .......... .......... .......... .......... .......... .......... ..........  [1080]
#KT318118.1_Bovine_coronavirus_isolate_BCoV/FRA/EPI/Caen/2013/08_spike_glycoprotein       .....E.... .......... ....W...A. .......... .......... .......... ......G... .......... .......... .......... .......... ..........  [1080]
#EU814648.1_Bovine_coronavirus_strain_438/06-TN-50_S_protein_(S)_gene_complete_cds        .....E.... .......... ....W...A. .......... .......... .......... ......G... .......... .......... .......... .......... ..........  [1080]
#KF169910.1_Bovine_coronavirus_isolate_SWE/02-2_spike_glycoprotein_(S)_gene_complete_cds  .....E.... .......... ....W...A. .......... .......... ........S. ......G... ....S..... .......... .......... .......... ..........  [1080]
#KF169913.1_Bovine_coronavirus_isolate_DEN/03-1_spike_glycoprotein_(S)_gene_complete_cds  .....E.... .......... ....W...A. .......... .......... .......... ......G... .......... .......... .......... .......... ..........  [1080]
#KF169934.1_Bovine_coronavirus_isolate_SWE/P/09-1_spike_glycoprotein_(S)_gene_complete_cds .....E.... .......... ....W...A. .......... .......... .......... ......G... ....S..... .......... .......... .......... ..........  [1080]
#EU019216_Bovine_coronavirus_Bubalus/Italy/179/07-11gene_spike_protein_(S)_4.9_kDs        .....E.... .......... ....W...A. .......... .......... .......... ......G... .......... .......... .......... .......... ..........  [1080]
#DQ389632.1_Bovine_coronavirus_isolate_KCD1_spike_glycoprotein_precursor_(S)_KOR          .....E.... .......... ....W...A. .......... .......... .......... ......G... D......... .......... .......... .......... ..........  [1080]
#DQ389658.1_Bovine_coronavirus_isolate_KWD17_spike_glycoprotein_precursor_(S)_KOR         .....E.... ...S...... ....W...A. .......... .......... .....I.... ......G... D......... .......... .......... .......... ..........  [1080]
#KF169932.1_Bovine_coronavirus_isolate_SWE/C/08-2_spike_glycoprotein_(S)_gene_complete_cds .....E.... .......... ....W...A. .......... .......... .......... ......G... .......... .......... .......... .......... ..........  [1080]
#KF169916.1_Bovine_coronavirus_isolate_DEN/05-1_spike_glycoprotein_(S)_gene_complete_cds  .....E.... .......... ....W...A. .......... .......... .......... ......G... ....S..... .......... .......... .......... ..........  [1080]
#KF169918.1_Bovine_coronavirus_isolate_DEN/05-3_spike_glycoprotein_(S)_gene_complete_cds  .....E.... .......... ....W...A. .......... .......... .......... ......G... ....S..... .......... .......... .......... ..........  [1080]
#EF193075.1_Bovine_coronavirus_strain_V270_1983_GER_spike_protein_gene                    .......... .......... ........A. .......... .......... .......... .......... .......... .......... ..T....... .......... ..........  [1080]
#DQ389637.1_Bovine_coronavirus_isolate_KCD6_spike_glycoprotein_precursor_(S)_gene-KOR     .....E.... .......... ....W...A. .......... .......... .......... ......G... D......... .......... .......... .......... ..........  [1080]
```

```
#KF169908.1_Bovine_coronavirus_isolate_SWE/C/92_spike_glycoprotein_(S)_gene_complete_cds     .....E.... .......... ....W...A. .......... .......... .......... ......G... .......... .......... .......... .......... ..........  [1080]
#D00731.1_Bovine_enteric_coronavirus_F15_1979_FRA_gene_for_spike_glycoprotein                .....E.... .......... ....W...A. .......... .......... .......... .......... .......... .......... .......... .......... ..........  [1080]
#ON792942_Bovine_coronavirus_strain_ABGEB0-54_S_gene_Ireland                                 .....E.... .......... ....W...A. .......... .......... .......... ......G... .......... .......... .......... .......... ..........  [1080]
#FJ938065.1Bovine_respiratory_coronavirus_AH187_complete_genome                              .....E.... .......... ....W...A. .......... .......... .......... ......G... .......... .......... .......... .......... ..........  [1080]
#MG757144.1_Bovine_coronavirus_isolate_strain_ICSA17-LBA_spike_protein_(S)                   .....E.... .......... ....W...A. .......... .......... .......... ......G... .......... .......... .......... ........F. ..........  [1080]
#MG757140_Bovine_coronavirus_isolate_ICSA16-LBA_2014_France                                  .....E.... .......... ....W...A. .......... .......... .......... ......G... .......... .......... .......... ........F. ..........  [1080]
#X2127-Marmot/Italy/193728-34/2023/CoV                                                       .....E.... .......... ....W...A. .......... .......... .......... ......G... .......... .......... .......... .......... ..........  [1080]
#X2128-Marmot/Italy/193728-35/2023/CoV                                                       .....E.... .......... ....W...A. .......... .......... .......... ......G... .......... .......... .......... .......... ..........  [1080]
```

HR-N domain

```
                                                                                                        1090       1100       1110       1120       1130       1140       1150       1160       1170       1180       1190
#U00735.2Bovine_coronavirus_strain_Mebus_complete_genome                                     ALEAQAQIDR LINGRLTALN VYVSQQLSDS TLVKFSAAQA MEKVNECVKS QSSRINFCGN GNHIISLVQN APYGLYFIHF SYVPTKYVTA KVSPGLCIAG DRGIAPKSGY FVNVNNTWMF  [1200]
#NC_003045_Bovine_coronavirus_isolate_BCoV-ENTSprotein_USA                                   .......... .......... A......... .......... .......... .......... .......... .......... .......... .......... .......... ..........  [1200]
#EF424615.1Bovine_coronavirus_E-AH65_complete_genome                                         .......... .......... A......... .......... .......... .......... .......... .......... .......... .......... .......... ..........  [1200]
#AF220295.1Bovine_coronavirus_strain_Quebec_complete_genome                                  .......... .......... .......... .......... .......... .......... .......... .......... .......... .......... .......... ..........  [1200]
#KT318118.1_Bovine_coronavirus_isolate_BCoV/FRA/EPI/Caen/2013/08_spike_glycoprotein          .......... .......... A......... .......... .......... .......... .......... .......... .......... .......... .......... ..........  [1200]
#EU814648.1_Bovine_coronavirus_strain_438/06-TN-50_S_protein_(S)_gene_complete_cds           .......... .......... A......... .......... .......... .......... .......... .......... .......... .......... .......... ..........  [1200]
#KF169910.1_Bovine_coronavirus_isolate_SWE/02-2_spike_glycoprotein_(S)_gene_complete_cds     .......... ...E...... A......... .......... .......... .......... .......... .......... .......... .......... .......... ..........  [1200]
#KF169913.1_Bovine_coronavirus_isolate_DEN/03-1_spike_glycoprotein_(S)_gene_complete_cds     .......... .......... A......... .......... .......... .......... .......... .......... .......... .......... .......... ..........  [1200]
#KF169934.1_Bovine_coronavirus_isolate_SWE/P/09-1_spike_glycoprotein_(S)_gene_complete_cds   .......... .......... A......... .......... .......... .......... .......... .......... .......... .......... .......... ..........  [1200]
#EU019216_Bovine_coronavirus_Bubalus/Italy/179/07-11gene_spike_protein_(S)_4.9_kDs           .......... ....... ... A......... .......... .......... .......... .......... .......... .......... .......... .......... ..........  [1200]
#DQ389632.1_Bovine_coronavirus_isolate_KCD1_spike_glycoprotein_precursor_(S)_KOR             .......... .......... A......... .......... .......... .......... .......... .......... .......... .......... .......... ..........  [1200]
#DQ389658.1_Bovine_coronavirus_isolate_KWD17_spike_glycoprotein_precursor_(S)_KOR            .......... .......... A......... .......... .......... .......... ........PH .......... .......... .......... .......... ..........  [1200]
#KF169932.1_Bovine_coronavirus_isolate_SWE/C/08-2_spike_glycoprotein_(S)_gene_complete_cds   .......... .......... A......... .......... .......... .......... .......... .......... .......... .......... .......... ..........  [1200]
#KF169916.1_Bovine_coronavirus_isolate_DEN/05-1_spike_glycoprotein_(S)_gene_complete_cds     .......... .......... A......... .......... .......... .......... .......... .......... .......... .......... .......... ..........  [1200]
#KF169918.1_Bovine_coronavirus_isolate_DEN/05-3_spike_glycoprotein_(S)_gene_complete_cds     .......... .......... A......... .......... .......... .......... .......... .......... .......... .......... .......... ..........  [1200]
#EF193075.1_Bovine_coronavirus_strain_V270_1983_GER_spike_protein_gene                       .......... .......... A......... .......... .......... .......... .......... .......... .......... .......... .......... ..........  [1200]
#DQ389637.1_Bovine_coronavirus_isolate_KCD6_spike_glycoprotein_precursor_(S)_gene-KOR        .......... .......... A......... .......... .......... .......... .......... .......... .......... .......... .......... ..........  [1200]
#KF169908.1_Bovine_coronavirus_isolate_SWE/C/92_spike_glycoprotein_(S)_gene_complete_cds     S......... .......... A......... .......... .......... .......... .......... .......... .......... .......... .......... ..........  [1200]
#D00731.1_Bovine_enteric_coronavirus_F15_1979_FRA_gene_for_spike_glycoprotein                .....R.... .....F.... A......... .......... .......... .......... .......... .......... .......... .......... .......... ..........  [1200]
#ON792942_Bovine_coronavirus_strain_ABGEB0-54_S_gene_Ireland                                 .......... .......... A......... .......... .......... .......... .......... .......... .......... .......... .......... ..........  [1200]
#FJ938065.1Bovine_respiratory_coronavirus_AH187_complete_genome                              .......... .......... A......... .......... .......... .......... .......... .......... .......... .......... G......... ......S...  [1200]
#MG757144.1_Bovine_coronavirus_isolate_strain_ICSA17-LBA_spike_protein_(S)                   .......... .......... A......... .......... .......... .......... .......... .......... .......... .......... .......... ..........  [1200]
#MG757140_Bovine_coronavirus_isolate_ICSA16-LBA_2014_France                                  .......... .......... A......... .......... .......... .......... .......... .......... .......... .......... .......... ..........  [1200]
#X2127-Marmot/Italy/193728-34/2023/CoV                                                       .......... .......... A......... .......... .......... .......... .......... .......... .......... .......... .......... ..........  [1200]
#X2128-Marmot/Italy/193728-35/2023/CoV                                                       .......... .......... A......... .......... .......... .......... .......... .......... .......... .......... .......... ..........  [1200]
```

HR-C domain

```
                                                                                                        1210       1220       1230       1240       1250       1260       1270       1280       1290       1300       1310
#U00735.2Bovine_coronavirus_strain_Mebus_complete_genome                                     TGSGYYYPEP ITGNNVVVMS TCAVNYTKAP DVMLNISTPN LHDFKEELDQ WFKNQTSVAP DLSLDYINVT FLDLQDEMNR LQEAIKVLNQ SYINLKDIGT YEYYVKWPWY VWLLIGFAGV  [1320]
#NC_003045_Bovine_coronavirus_isolate_BCoV-ENTSprotein_USA                                   .......... .......... .......... .......... .PY....... .......... .......... .......... .......... .......... .......... ..........  [1320]
#EF424615.1Bovine_coronavirus_E-AH65_complete_genome                                         .......... .......... .......... .......... .P........ .......... .......... .......... .......... .......... .......... ..........  [1320]
#AF220295.1Bovine_coronavirus_strain_Quebec_complete_genome                                  .......... .......... .......... .......... .......... .......... .......... .......... .......... .......... .......... ..........  [1320]
#KT318118.1_Bovine_coronavirus_isolate_BCoV/FRA/EPI/Caen/2013/08_spike_glycoprotein          .......... .......... .......... .......... .P........ ....H..... N......... .......... .......... .......... .......... ..........  [1320]
#EU814648.1_Bovine_coronavirus_strain_438/06-TN-50_S_protein_(S)_gene_complete_cds           .......... .......... ...F...... .......... .P........ .........S N......... .......... .......... .......... .......... ..........  [1320]
#KF169910.1_Bovine_coronavirus_isolate_SWE/02-2_spike_glycoprotein_(S)_gene_complete_cds     .......... .......... .......... .........K .P........ .......... N......... .......... .......... .......... .......... ..........  [1320]
#KF169913.1_Bovine_coronavirus_isolate_DEN/03-1_spike_glycoprotein_(S)_gene_complete_cds     .......... .......... .......... .......... .P........ .......... N......... .......... .......... .......... .......... I.........  [1320]
#KF169934.1_Bovine_coronavirus_isolate_SWE/P/09-1_spike_glycoprotein_(S)_gene_complete_cds   .......... .......... .......... .......I.. .P........ .......... N......... .......... .......... .......... .......... ..........  [1320]
#EU019216_Bovine_coronavirus_Bubalus/Italy/179/07-11gene_spike_protein_(S)_4.9_kDs           .......... .......... .......... .......... .P........ .......... N......... .......... .......... .......... .......... ..........  [1320]
#DQ389632.1_Bovine_coronavirus_isolate_KCD1_spike_glycoprotein_precursor_(S)_KOR             .......... .......... .......... .......... .P........ .......... .......... .......I.. .......... .......... .......... ....V.....  [1320]
#DQ389658.1_Bovine_coronavirus_isolate_KWD17_spike_glycoprotein_precursor_(S)_KOR            .......... .......... .......... .......... .P........ ....R..... ......K... .......... .......... .......... .......... ..........  [1320]
#KF169932.1_Bovine_coronavirus_isolate_SWE/C/08-2_spike_glycoprotein_(S)_gene_complete_cds   .......... .......... .......... .......I.. .P........ .......... N......... ....H..... .......... .......... .......... ..........  [1320]
#KF169916.1_Bovine_coronavirus_isolate_DEN/05-1_spike_glycoprotein_(S)_gene_complete_cds     .......... .......... .......... .....T.... .P........ .......... N......... .......... .......... .......... .......... ..........  [1320]
#KF169918.1_Bovine_coronavirus_isolate_DEN/05-3_spike_glycoprotein_(S)_gene_complete_cds     .......... .......... .......... .....T.... .P........ .......... N......... .......... .......... .......... .......... ..........  [1320]
#EF193075.1_Bovine_coronavirus_strain_V270_1983_GER_spike_protein_gene                       .......... .......... .......... .......... .......... .......... .......... .......... .......... .......... .......... ..........  [1320]
#DQ389637.1_Bovine_coronavirus_isolate_KCD6_spike_glycoprotein_precursor_(S)_gene-KOR        .......... .......... .......... .......... .P........ .......... .......... .......... .......... .......... .......... ..........  [1320]
#KF169908.1_Bovine_coronavirus_isolate_SWE/C/92_spike_glycoprotein_(S)_gene_complete_cds     .......... .......... .......... .......... .P........ .......... .......... .......... .......... .......... .......... ..........  [1320]
#D00731.1_Bovine_enteric_coronavirus_F15_1979_FRA_gene_for_spike_glycoprotein                .......... .......... .......... .......... .P........ .......... .......... ......L... .......... .......... .......... ..........  [1320]
#ON792942_Bovine_coronavirus_strain_ABGEB0-54_S_gene_Ireland                                 .......... .......... .......... .......... .P........ ....H..... N......... .......... .......... .......... .......... ..........  [1320]
#FJ938065.1Bovine_respiratory_coronavirus_AH187_complete_genome                              .......... .......... .......... .......... .P........ .......... .......... .......... .......... .......... .......... ..........  [1320]
#MG757144.1_Bovine_coronavirus_isolate_strain_ICSA17-LBA_spike_protein_(S)                   .......... .......... .......... .......... .P........ ....H..... N......... .......... .......... .......... .......... ..........  [1320]
#MG757140_Bovine_coronavirus_isolate_ICSA16-LBA_2014_France                                  .......... .......... .......... .......... .P........ ....H..... N......... .......... .......... .......... .......... ..........  [1320]
#X2127-Marmot/Italy/193728-34/2023/CoV                                                       .......... .......... .......... .......... .P........ ....H..... N......... .......... .......... .......... .......... ..........  [1320]
#X2128-Marmot/Italy/193728-35/2023/CoV                                                       .......... .......... .......... .......... .P........ ....H..... N......... .......... .......... .......... .......... ..........  [1320]
```

```
                                                                                                        1330       1340       1350       1360
#U00735.2Bovine_coronavirus_strain_Mebus_complete_genome                                     AMLVLLFFIC CCTGCGTSCF KICGGCCDDY TGHQELVIKT SHDD* [1365]
#NC_003045_Bovine_coronavirus_isolate_BCoV-ENTSprotein_USA                                   .......... .......... .K........ .......... ..E.. [1365]
#EF424615.1Bovine_coronavirus_E-AH65_complete_genome                                         .......... .......... .K........ .......... ..E.. [1365]
#AF220295.1Bovine_coronavirus_strain_Quebec_complete_genome                                  .......... .......... .......... .......... ..... [1365]
#KT318118.1_Bovine_coronavirus_isolate_BCoV/FRA/EPI/Caen/2013/08_spike_glycoprotein          .......... .......... .K........ .......... ..... [1365]
#EU814648.1_Bovine_coronavirus_strain_438/06-TN-50_S_protein_(S)_gene_complete_cds           .......... .......... .......... .......... ..... [1365]
#KF169910.1_Bovine_coronavirus_isolate_SWE/02-2_spike_glycoprotein_(S)_gene_complete_cds     .......... .......... .K........ .......... ..... [1365]
#KF169913.1_Bovine_coronavirus_isolate_DEN/03-1_spike_glycoprotein_(S)_gene_complete_cds     .......... .......... .K.....E.. I.Y....... ..... [1365]
#KF169934.1_Bovine_coronavirus_isolate_SWE/P/09-1_spike_glycoprotein_(S)_gene_complete_cds   .......... .......... .K........ .......... ..... [1365]
#EU019216_Bovine_coronavirus_Bubalus/Italy/179/07-11gene_spike_protein_(S)_4.9_kDs           .......... .......... .K........ .......... ..... [1365]
#DQ389632.1_Bovine_coronavirus_isolate_KCD1_spike_glycoprotein_precursor_(S)_KOR             .......... .......... .K........ .......... ..... [1365]
#DQ389658.1_Bovine_coronavirus_isolate_KWD17_spike_glycoprotein_precursor_(S)_KOR            .......... ........R. .......... A......... ..... [1365]
#KF169932.1_Bovine_coronavirus_isolate_SWE/C/08-2_spike_glycoprotein_(S)_gene_complete_cds   .......... .......... .K........ .......... ..... [1365]
#KF169916.1_Bovine_coronavirus_isolate_DEN/05-1_spike_glycoprotein_(S)_gene_complete_cds     .......... .......... .K........ .......... ..... [1365]
```

```
#KF169918.1_Bovine_coronavirus_isolate_DEN/05-3_spike_glycoprotein_(S)_gene_complete_cds      .......... .......... .K........ .......... ..... [1365]
#EF193075.1_Bovine_coronavirus_strain_V270_1983_GER_spike_protein_gene                        .......... .......... .......... .......... ..... [1365]
#DQ389637.1_Bovine_coronavirus_isolate_KCD6_spike_glycoprotein_precursor_(S)_gene-KOR         .......... .......... .K........ .......... ..... [1365]
#KF169908.1_Bovine_coronavirus_isolate_SWE/C/92_spike_glycoprotein_(S)_gene_complete_cds      .......... .......... .K........ .......... ..... [1365]
#D00731.1_Bovine_enteric_coronavirus_F15_1979_FRA_gene_for_spike_glycoprotein                 .......... .......... .K........ .......... ..... [1365]
#ON792942_Bovine_coronavirus_strain_ABGEB0-54_S_gene_Ireland                                  .......... .......... .K........ .......... ..... [1365]
#FJ938065.1Bovine_respiratory_coronavirus_AH187_complete_genome                               .......... .......... .K........ .......... ..E.. [1365]
#MG757144.1_Bovine_coronavirus_isolate_strain_ICSA17-LBA_spike_protein_(S)                    .......... ....S..... .K........ .......... ..... [1365]
#MG757140_Bovine_coronavirus_isolate_ICSA16-LBA_2014_France                                   .......... .......... .K........ .......... ..... [1365]
#X2127-Marmot/Italy/193728-34/2023/CoV                                                        .......... .......... .K........ .......... ..E.. [1365]
#X2128-Marmot/Italy/193728-35/2023/CoV                                                        .......... .......... .K........ .......... ..E.. [1365]
```

Table S2- Molecular analysis of the amino acid (aa) sequences of the complete S protein of Italian marmots and reference and field B-CoV. Immune reactive domains (red) and regions important for virus-cell interactions (blue) are shown in boxes. Particular aa changes suggested as being linked to virulence factors (red), and enteric (green) and respiratory (blue) tropism are indicated by arrows [25]. Amino acid changes observed only in the marmots sequences are evidenced in yellow.

HE protein

```
                                                     Signal peptide  Membrane proximal              Esterase                                              Lectin
                                                             10         20         30         40         50         60         70         80         90        100        110        120
#U00735.2_Bovine_coronavirus_strain_Mebus_USA              MFLLLRFVLV SCIIGSLGFD NPPTNVVSHL NGDWFLFGDS RSDCNHVVNT NPRNYSYMDL NPALCDSGKI SSKAGNSIFR SFHFTDFYNY TGEGQQIIFY EGVNFTPYHA FKCTTSGSND [120]
#NC_003045_Bovine_coronavirus_isolate_BCoV-ENT_USA         ....P..... .......... .......... .......... ........T. .......... .....G.... .......... .......... .......... .......... .......... [120]
#EF424615._Bovine_coronavirus_E-AH65_complete_genome       ....P..... .......... .......... .......... ........T. .......... .....G.... .A........ .......... .......... .......... .......... [120]
#AF220295_Bovine_coronavirus_strain_Quebec_Canada          .......... .......... .......... .......... .......... .......... .......... .......... .......... .......... ..L....... .......... [120]
#EU019216_Bovine_coronavirus_Bubalus/Italy/179/07-11_Italy ....P..... .......... .......... .......... ........T. .......... .......... .......... .......... .......... .......H.. .......... [120]
#DQ994168.1:6-1275_Bovine_coronavirus_isolate_KWD17_Korea  ....P.L... .......... .......... .......... ........T. .......... .....G.... .......... .......... .......... .......... .......... [120]
#ON792942.1_Bovine_coronavirus_strain_ABGEB0-54_2019_Ireland ....P..... .......... .......... .......... ........T. .......... .......... .......... .......... .......... .......... .......... [120]
#ON792962_Bovine_coronavirus_strain_ABGEBB-129_Ireland     ....P..... .......... .......... .......... ........T. .......... .......... .......... .......... .......... .......... .......... [120]
#FJ938065.1_Bovine_respiratory_coronavirus_AH187_USA_2000  ....P..... .......... .......... .......... ........T. .......... .....G.... .A........ .......... .......... .......... .......... [120]
#MG757140_Bovine_coronavirus_isolate_ICSA16-LBA_2014_France ....P..... ........-Y .......... .......... ........T. .......... .......... .......... .......... .......... .......... .......... [120]
#MG757141_Bovine_coronavirus_isolate_ICSA-pool-EN_France   ....P..... .......... .......... .......... ........T. .......... .......... .......... .......... .......... .......... .......... [120]
#KX982264_Bovine_coronavirus_isolate_BCoV_2014_13_France   ....P..... .......... .......... .......... ........T. .......... .......... .......... .......... .......... .......H.. .......... [120]
#Marmot/Italy/193728-35/2023/CoV                           ....P..... .......... .......... .......... ........T. .......... .......... .......... .......... .......... .......H.. .......... [120]
#Marmot/Italy/193728-34/2023/CoV                           ....P..... .......... .......... T......... ........T. .......... .......... .......... .......... .......... .......H.. .......... [120]
#OR367715_Bovine_coronavirus_isolate_BCoV/XJ21/CHN/2023_China ....P..... .......... .......... .......... ........T. .......... .....G.... .......... .......... .......... .......... .......... [120]
#MN982199_Bovine_coronavirus_isolate_BCOV-China/SWUN/A10/2018 ....P..... .......... .......... .......... ........T. .......... .......... .......... .......... .......... ........   . [120]

                                                                                         Lectin                                                      R3-loop        Lectin
                                                                130        140        150        160        170        180        190        200        210  RBS   220        230
#U00735.2_Bovine_coronavirus_strain_Mebus_USA              IWMQNKGLFY TQVYKNMAVY RSLTFVNVPY VYNGSAQSTA LCKSGSLVLN NPAYIAREAN FGDYYYKVEA DFYLSGCDEY IVPLCIFNGK F----LSNTK YYDDSQYYFN KDTGVIYGLN [240]
#NC_003045_Bovine_coronavirus_isolate_BCoV-ENT_USA         .......... .......... .......... .......... .......... .......... .......... .......... .......... .----..... .......... .......... [240]
#EF424615._Bovine_coronavirus_E-AH65_complete_genome       .......... .......... .......... .......... .......... .......... .......... .......... .......... .----..... .......... .......... [240]
#AF220295_Bovine_coronavirus_strain_Quebec_Canada          .......... .......... .......... .......... .......... .......... .......... .......... .......... .----..... .......... .......... [240]
#EU019216_Bovine_coronavirus_Bubalus/Italy/179/07-11_Italy .......... .......... .......... ......P... .......... .......... .......... .......... .........N .----..... .......... .......... [240]
#DQ994168.1:6-1275_Bovine_coronavirus_isolate_KWD17_Korea  .......... .......... .......... .......... .......... .......... .......... .......... .......... .----..... .......... .......... [240]
#ON792942.1_Bovine_coronavirus_strain_ABGEB0-54_2019_Ireland .......... .......... .......... ......P... .......... .......... L......... .......... .......... .----..S.. .......... .......... [240]
#ON792962_Bovine_coronavirus_strain_ABGEBB-129_Ireland     .......... .......... .......... ......P... .......... .......... L......... .......... .......... .----..S.. .......... .......... [240]
#FJ938065.1_Bovine_respiratory_coronavirus_AH187_USA_2000  .......... .......... .......... .......... .......... .......... .......... .......... .......... .----..... .......... .......... [240]
#MG757140_Bovine_coronavirus_isolate_ICSA16-LBA_2014_France .......... .......... .......... .......... .......... .......... .......... .......... .......... .----..... .......... .......... [240]
#MG757141_Bovine_coronavirus_isolate_ICSA-pool-EN_France   .......... .......... .......... .......... .......... .......... .......... .......... .......... .----..... .......... .......... [240]
#KX982264_Bovine_coronavirus_isolate_BCoV_2014_13_France   .......... .......... .......... ......P... .......... .......... .......... .......... .......... .----..... .......... .......... [240]
#Marmot/Italy/193728-35/2023/CoV                           .......... .......... .......... ......P... .......... .....K.... .......... .......... .......... .----..... .......... .......... [240]
#Marmot/Italy/193728-34/2023/CoV                           .......... .......... .......... ......P... .......... .....K.... .......... .......... .......... .----..... .......... .......... [240]
#OR367715_Bovine_coronavirus_isolate_BCoV/XJ21/CHN/2023_China .......... .......... .......... .......... .......... .......... .......... .......... .......... .----..... .......... .......... [240]
#MN982199_Bovine_coronavirus_isolate_BCOV-China/SWUN/A10/2018 .......... .......... .......... ......A... .......... .......... V.....A... .......... .......... .KATV..... .......... .......... [240]

                                                            R4-loop             Lectin                              Esterase                                       Membrane proximal
                                                             240        250        260        270        280        290        300        310        320        330        340        350
#U00735.2_Bovine_coronavirus_strain_Mebus_USA              STETITTGFD FNCHYLVLPS GNYLAISNEL LLTVPTKAIC LNKRKDFTPV QVVDSRWNNA RQSDNMTAVA CQPPYCYFRN STTNYVGVYD INHGDAGFTS ILSGLLYDSP CFSQQGVFRY [360]
#NC_003045_Bovine_coronavirus_isolate_BCoV-ENT_USA         .......... .......... .......... .......... .......... .......... .......... .......... .......... .......... .......... .......... [360]
#EF424615._Bovine_coronavirus_E-AH65_complete_genome       .......... .......... .......... .......... .......... .......... .......... .......... .......... .......... .......... .......... [360]
#AF220295_Bovine_coronavirus_strain_Quebec_Canada          .......... .......... .......... .......... .......... .......... .......... .......... .......... .......... .......... .......... [360]
#EU019216_Bovine_coronavirus_Bubalus/Italy/179/07-11_Italy .......... .......... .......... .......... .......... .......... .......... .......... .......... .......... .......... .......... [360]
#DQ994168.1:6-1275_Bovine_coronavirus_isolate_KWD17_Korea  .......... .......... .......... .......... .......... .......... .......... .......... .......... .......... .......... .......... [360]
#ON792942.1_Bovine_coronavirus_strain_ABGEB0-54_2019_Ireland A.Q....... .......... .......... .......... .......... .......... .......... .......... .......... .......... .......... .......... [360]
#ON792962_Bovine_coronavirus_strain_ABGEBB-129_Ireland     A.Q....... .......... .......... .......... .......... .......... .......... .......... .......... .......... .......... .......... [360]
#FJ938065.1_Bovine_respiratory_coronavirus_AH187_USA_2000  .......... .......... .......... .......... .......... .......... .......... .......... .......... .......... .......... .......... [360]
#MG757140_Bovine_coronavirus_isolate_ICSA16-LBA_2014_France .......... .......... .......... .......... .......... .......... .......... .......... .......... .......... .......... .......... [360]
#MG757141_Bovine_coronavirus_isolate_ICSA-pool-EN_France   .......... .......... .......... .......... .......... .......... .......... .......... .......... .......... .......... .......... [360]
#KX982264_Bovine_coronavirus_isolate_BCoV_2014_13_France   .......... .......... .......... .......... .......... .......... .......... .......... .......... .......... .......... .......... [360]
#Marmot/Italy/193728-35/2023/CoV                           A.Q....... .......... .......... .......... .......... .......... .......... .......... .......... .......... .......... .......... [360]
#Marmot/Italy/193728-34/2023/CoV                           A.Q....... .......... .......... .......... .......... .......... .......... .......... .......... .......... .......... .......... [360]
#OR367715_Bovine_coronavirus_isolate_BCoV/XJ21/CHN/2023_China .......... .......... .......... .......... .......... .......... .......... .......... .......... .......... .......... .......... [360]
#MN982199_Bovine_coronavirus_isolate_BCOV-China/SWUN/A10/2018 .......... .......... .......... .......... .......... .......... .......... .......... .......... .......... .......... .......... [360]

                                                                 Membrane proximal
                                                             360        370        380        390        400        410        420
#U00735.2_Bovine_coronavirus_strain_Mebus_USA              DNVSSVWPLY SYGRCPTAAD INTPDVPICV YDPLPLILLG ILLGVAVIII VVLLLYFMVD NGTRLHDA* [429]
#NC_003045_Bovine_coronavirus_isolate_BCoV-ENT_USA         .......... P......... .......... .....I.... .......... .......... ......... [429]
#EF424615._Bovine_coronavirus_E-AH65_complete_genome       .......... P......... .......... .....I.... .......... .......... ......... [429]
#AF220295_Bovine_coronavirus_strain_Quebec_Canada          .......... .......... .......... .......... .......... .......... ......... [429]
#EU019216_Bovine_coronavirus_Bubalus/Italy/179/07-11_Italy .......... P......... .......... .....I.... .......... .......... ......... [429]
#DQ994168.1:6-1275_Bovine_coronavirus_isolate_KWD17_Korea  .......... P......... .......... .....I.... .......... .......... ......... [429]
#ON792942.1_Bovine_coronavirus_strain_ABGEB0-54_2019_Ireland .....I.... P......... .......... .....I.... .......... .......... ......... [429]
#ON792962_Bovine_coronavirus_strain_ABGEBB-129_Ireland     .....I.... P......... .......... .....I.... .......... .......... ......... [429]
#FJ938065.1_Bovine_respiratory_coronavirus_AH187_USA_2000  .......... .......... .......... .....I.... .......... .......... ......... [429]
#MG757140_Bovine_coronavirus_isolate_ICSA16-LBA_2014_France .....I.... P......... .......... .....I.... .......... .......... ......... [429]
#MG757141_Bovine_coronavirus_isolate_ICSA-pool-EN_France   .....I.... P......... .......... .....I.... .......... .......... ......... [429]
#KX982264_Bovine_coronavirus_isolate_BCoV_2014_13_France   ..I....... P......... .......... .....I.... .......... .......... ......... [429]
#Marmot/Italy/193728-35/2023/CoV                           .......... P......... .......... .....I.... .......... .......... ......... [429]
#Marmot/Italy/193728-34/2023/CoV                           .......... P......... .......... .....I.... .......... .......... ......... [429]
#OR367715_Bovine_coronavirus_isolate_BCoV/XJ21/CHN/2023_China .......... P......... .......... .....I.... .......... .......... ......... [429]
#MN982199_Bovine_coronavirus_isolate_BCOV-China/SWUN/A10/2018 .......... P......... ...H...... .....I.... .......... .......... ......... [429]
```

Table S3 - Molecular analysis of the amino acid sequences (aa) of the complete HE protein of Italian marmots and reference and field B-CoVs. The domains of the HE protein, the R3 and R4 loops and the receptor-binding domain (RBD) are highlighted in boxes [26]. Amino acid changes observed only in the marmot sequences are highlighted in yellow.
